# An EHBP-1-SID-3-DYN-1 axis promotes membranous tubule fission during endocytic recycling

**DOI:** 10.1371/journal.pgen.1008763

**Published:** 2020-05-08

**Authors:** Jinghu Gao, Linyue Zhao, Qian Luo, Shuyao Liu, Ziyang Lin, Peixiang Wang, Xin Fu, Juan Chen, Hongjie Zhang, Long Lin, Anbing Shi

**Affiliations:** 1 Department of Biochemistry and Molecular Biology, School of Basic Medicine, Tongji Medical College, Huazhong University of Science and Technology, Wuhan, Hubei, China; 2 Faculty of Health Sciences, University of Macau, Avenida da Universidade, Taipa, Macau SAR, China; 3 Institute for Brain Research, Huazhong University of Science and Technology, Wuhan, Hubei, China; 4 Key Laboratory of Neurological Disease of National Education Ministry, Tongji Medical College, Huazhong University of Science and Technology, Wuhan, Hubei, China; University of California San Diego, UNITED STATES

## Abstract

The ACK family tyrosine kinase SID-3 is involved in the endocytic uptake of double-stranded RNA. Here we identified SID-3 as a previously unappreciated recycling regulator in the *Caenorhabditis elegans* intestine. The RAB-10 effector EHBP-1 is required for the endosomal localization of SID-3. Accordingly, animals with loss of SID-3 phenocopied the recycling defects observed in *ehbp-1* and *rab-10* single mutants. Moreover, we detected sequential protein interactions between EHBP-1, SID-3, NCK-1, and DYN-1. In the absence of SID-3, DYN-1 failed to localize at tubular recycling endosomes, and membrane tubules breaking away from endosomes were mostly absent, suggesting that SID-3 acts synergistically with the downstream DYN-1 to promote endosomal tubule fission. In agreement with these observations, overexpression of DYN-1 significantly increased recycling transport in SID-3-deficient cells. Finally, we noticed that loss of RAB-10 or EHBP-1 compromised feeding RNAi efficiency in multiple tissues, implicating basolateral recycling in the transport of RNA silencing signals. Taken together, our study demonstrated that in *C*. *elegans* intestinal epithelia, SID-3 acts downstream of EHBP-1 to direct fission of recycling endosomal tubules in concert with NCK-1 and DYN-1.

## Author summary

After endocytic uptake, a recycling transport system is deployed to deliver endocytosed macromolecules, fluid, membranes, and membrane proteins back to the cell surface. This process is essential for a series of biological processes such as cytokinesis, cell migration, maintenance of cell polarity, and synaptic plasticity. Recycling endosomes mainly consist of membrane tubules and often undergo membrane fission to generate vesicular carriers, which mediates the delivery of cargo proteins back to the plasma membrane. Previous studies suggested that RAB-10 and its effector protein EHBP-1 function jointly to generate and maintain recycling endosomal tubules. However, the mechanism coupling recycling endosomal tubulation and membrane fission remains elusive. Here, we identified SID-3 as a new interactor of EHBP-1. EHBP-1 is required for the endosomal localization of SID-3 and initiates a protein interaction cascade involving SID-3, NCK-1, and DYN-1/dynamin. We also found that SID-3 functions downstream of EHBP-1 to encourage membrane scission, and that ectopic expression of DYN-1 improves recycling transport in SID-3-depleted cells. Our findings revealed EHBP-1 as a point of convergence between RAB-10-mediated endosomal tubulation and SID-3-assisted membrane tubule fission during endocytic recycling.

## Introduction

Recycling endosomes receive cargos from sorting endosomes and eventually deliver them to the cell surface [1, 2]. Membrane-associated cargos destined for the plasma membrane are concentrated in tubular membrane carriers for proper sorting away from the lumenal content [2–4]. Tubular recycling carrier formation involves a variety of regulators including RAB-10/Rab10, EHBP-1/EHBP1, RME-1/EHD1, the actin cytoskeleton, and the exocyst [5–9]. In the *C*. *elegans* intestine, RAB-10 functions in sorting endosomes and coordinates basolateral recycling transport upstream of RME-1 [10, 11]. In particular, RAB-10 directs the recycling of cargo proteins internalized via CIE (clathrin-independent endocytosis), such as hTAC (the alpha-chain of the human IL2 receptor) [10]. EHBP-1 was characterized as a specific effector of RAB-10 [12], and the depletion of EHBP-1 yields phenotypes resembling those of *rab-10* mutants. Also, mammalian EHBP1 was reported to associate with EHD1 and EHD2 to modulate GLUT4 recycling in adipocytes [13, 14]. A recent study in *C*. *elegans* further showed that the EHBP-1 calponin homology (CH) domain preferentially associates with F-actin, and thus assists the tubulation of recycling endosomes [5].

Mammalian recycling endosomes consist of membrane tubules and discrete vesicles [1]. Similarly, the recycling endosomal network in the *C*. *elegans* intestine is highly tubular [15]. Lack of RAB-10 or EHBP-1 leads to the collapse of the endosomal meshwork, suggesting that RAB-10 and EHBP-1 function jointly to generate and/or maintain endosomal tubules [10, 12]. In agreement with this observation, RAB-10 is located at the tips of newly formed tubules and functions in concert with the exocyst to direct the extension and tethering of membrane tubules [8]. Like other types of endosomes, recycling endosomes undergo fission to generate tubular carriers [9]. However, the mechanism coordinating recycling endosomal tubulation and membrane fission has been a long-standing mystery, primarily because the link between tubule biogenesis/maintenance and subsequent scission is unknown.

ACK (activated CDC42-associated kinase) is a non-receptor tyrosine kinase that was initially identified as a CDC42 effector protein [16]. ACK family kinases contain an N-terminal tyrosine kinase domain, an SH3 (src homology 3) domain, a CRIB (CDC42/RAC interacting binding) domain, and a highly variable C-terminal region [17]. ACK is found in clathrin-coated pits [18] and interacts with the clathrin heavy chain [19]. Several ACK-interacting proteins have been reported, including the adaptor protein NCK [20–22]. During sperm differentiation, ACK is required for the subcellular distribution of Dock, the *Drosophila* homolog of NCK [22]. NCK harbors three SH3 domains and a C-terminal SH2 domain [23], and the interaction between ACK and NCK is mediated by the SH2 domain [24]. Importantly, NCK was found to interact with dynamin via the third SH3 domain [25], suggesting that NCK and dynamin could function in the same pathway.

The *C*. *elegans* genome encodes two ACK family tyrosine kinases: SID-3 and ARK-1 (ACK related kinase-1). Phylogenetic and functional analysis suggested that SID-3 is the sole homolog of the mammalian ACK [26]. SID-3 is broadly expressed and localizes to cytosolic puncta [26]. Functional analysis indicated that SID-3 and its mammalian homolog ACK encourage double-stranded RNA (dsRNA) uptake, and this efficacy appears to involve clathrin-mediated endocytosis [19, 26]. In the present study, we identified SID-3 as a novel interactor of EHBP-1 and highlighted the necessity of EHBP-1 for the endosomal localization of SID-3. Loss of SID-3 led to a severe defect in the recycling of the clathrin-independent cargo hTAC-GFP. Our results also suggested that SID-3 functions downstream of RAB-10 to facilitate recycling in concert with NCK-1/NCK and DYN-1/dynamin. Accordingly, in *sid-3* mutants, the detachment of membrane tubules from recycling endosomes largely did not occur, and the overexpression of DYN-1 effectively restored the recycling of hTAC-GFP. Together, our findings demonstrate that EHBP-1 represents a point of convergence between RAB-10-directed endosomal tubulation and SID-3-coordinated membrane fission and promotes tubular recycling carrier formation.

## Results

### Loss of SID-3 causes endocytic recycling defects

**Fig 1:**
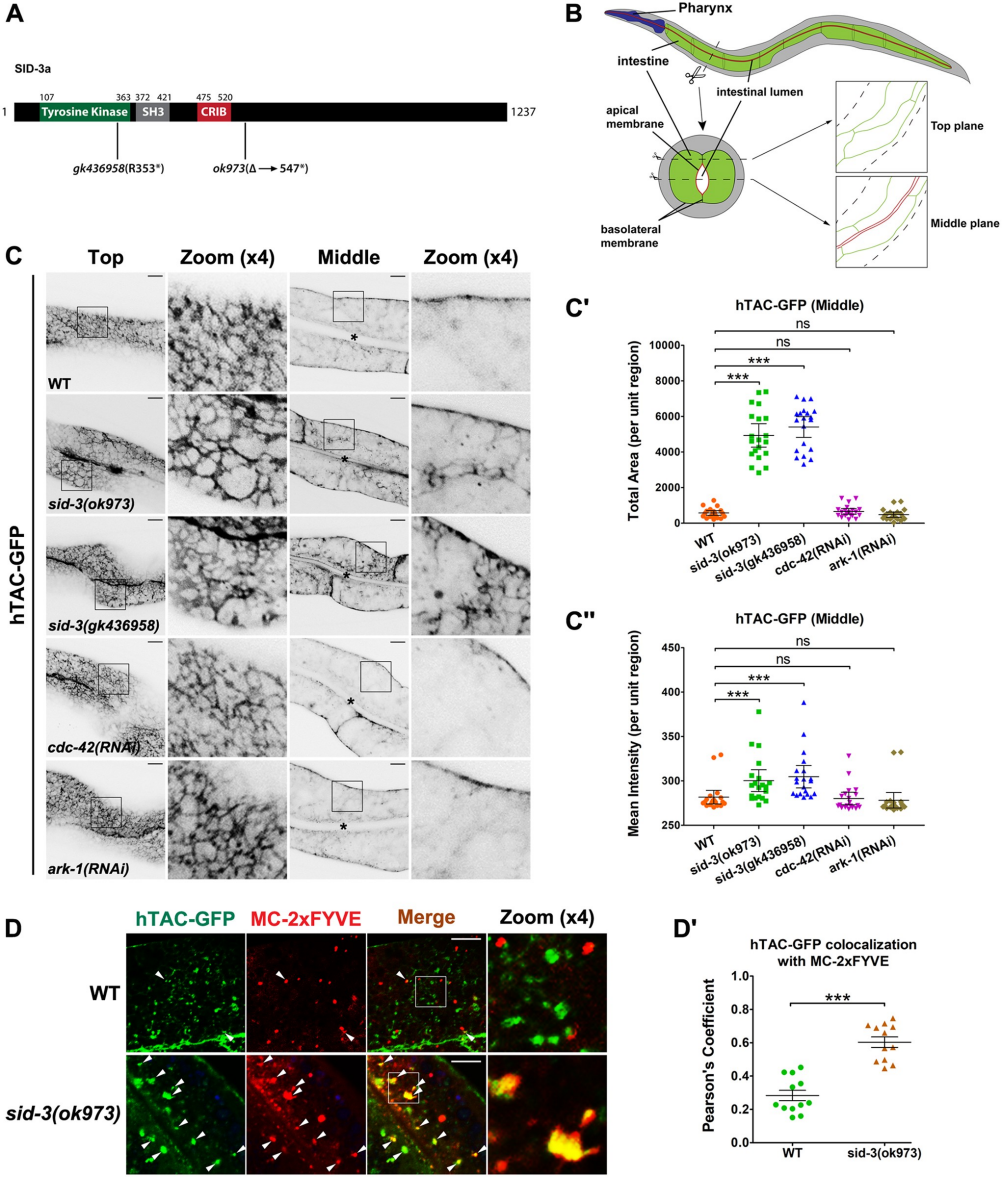
SID-3 is required for sustaining recycling transport in intestinal epithelia. (**A**) Domain architecture of SID-3a. SID-3a contains an N-terminal tyrosine kinase domain, an SH3 (src homology 3) domain, a CRIB (CDC42/RAC interacting binding) domain, and a highly variable C-terminal region. *sid-3* mutant alleles and the corresponding sequence changes were indicated. Δ: deletion; *: stop codon. (**B**) A diagram of the worm intestine, indicating the apical and basal membrane and the top and middle focal planes of the confocal microscopy. Imaging plane close to the basal membrane is defined as "Top", and the imaging plane where the apical membrane and lumen can be observed is defined as "Middle". (**C-C″**) Confocal images of the worm intestinal cells expressing GFP-tagged recycling cargo protein, the IL-2 receptor alpha chain (hTAC-GFP). In *sid-3(ok973)* and *sid-3(gk436958)* mutants, hTAC-GFP significantly accumulated in the intracellular structures. In contrast, the distribution of hTAC-GFP-positive structures in CDC-42- or ARK-1-deficient cells had no significant aberration. Black asterisks in the panels indicate intestinal lumen. Error bars are 95% CIs (n = 20 each, 10 animals of each genotype were sampled in whole-cell regions of two intestinal cells). Asterisks indicate the significant differences in the Mann-Whitney test (***p<0.001, **p<0.01, ns: no significance). (**D-Dꞌ**) Confocal image showing colocalization between hTAC-GFP and mCherry-tagged early endosome marker EEA-1-2xFYVE in the intestinal cells. Compared with the wild-type animals, the overlap level of hTAC-GFP with 2xFYVE has significantly improved in *sid-3(ok973)* mutants. Arrowheads indicate structures labeled by both hTAC-GFP and mCherry-2xFYVE. Pearson’s correlation coefficients for GFP and mCherry signals are calculated, error bars are 95% CIs (n = 12 animals, ***p<0.001). Scale bars, 10 μm. See S1 Table for quantitative data in this figure.

In the current study, we focused on basolateral recycling transport in the *C*. *elegans* intestine, a polarized epithelial tube (Fig 1B). The apical membrane faces the digestive lumen and is responsible for nutrient uptake. The basolateral membrane faces the pseudocoelom and is responsible for the exchange of molecules between the intestine and other tissues. In the *C*. *elegans* intestine, the basolateral sorting endosomes are enriched in the recycling regulator RAB-10 [8, 12]. RAB-10 is predominantly involved in the recycling of CIE cargos [10, 27]. Through its effectors, EHBP-1 and CNT-1, RAB-10 bridges endosomes with F-actin and manipulates the level of endosomal PI(4,5)P2 (phosphatidylinositol 4,5-bisphosphate) to ensure proper recycling transport [5, 12]. However, the mechanistic processes underlying recycling regulation are still poorly understood. To address this, we conducted a genome-wide RNAi screen to isolate genes that when silenced cause overaccumulation of the CIE cargo hTAC in the deep cytosol of intestinal epithelia (please see Materials and methods for details). After a preliminary screen and subsequent validation, we found that knockdown of SID-3/ACK resulted in prominent intracellular deposition of hTAC-GFP.

To confirm the role of SID-3 in recycling, we employed two mutant alleles: *sid-3(ok973)* and *sid-3(gk436958)*. *sid-3(ok973)* harbors a premature termination codon at amino acid site 547 that leads to a 690-amino acid deletion at the C-terminus. *sid-3(gk436958)* carries a point-nonsense mutation at site 353, resulting in a deletion that spans part of the tyrosine kinase domain and the entire C-terminal region (Fig 1A). Through confocal stack imaging of intestinal epithelial cells from intact living animals (Fig 1B), we found that, in both *sid-3* mutants, the number and size of hTAC-GFP-labeled intracellular structures increased significantly (~9-fold increase in total area) (Fig 1C–1C''). This phenotype is typical of basolateral recycling mutants [5, 12, 28–30]. We further assayed the accumulation of FGT-1, the worm homolog of another clathrin-independent recycling cargo, GLUT1 [8], and observed that SID-3 deficiency led to substantial intracellular aggregation of FGT-1-positive structures (~7.8-fold increase in total area) (S1A and S1A' Fig). We also examined the well-defined clathrin-dependent recycling cargo hTfR (human transferrin receptor) [10, 31]. Loss of SID-3 caused accumulation of hTfR-GFP on the plasma membrane (~11.5-fold increase in total area) (S1B and S1B' Fig). Likewise, SID-3-depleted cells exhibited poor endocytic uptake of the clathrin-dependent retrograde cargo MIG-14 (~13.3-fold increase in Top total area) (S1B and S1B' Fig) [32]. Hence, our results indicate that, in addition to its previously described role in clathrin-dependent endocytosis [18, 19], SID-3 is involved in the regulation of the recycling of clathrin-independent cargos.

Next, to assess where SID-3 functions in the recycling pathway, we compared the localization of hTAC-GFP with that of an early endosome marker, 2xFYVE [5]. hTAC-GFP was rarely localized in 2xFYVE-labeled early endosomes in the wild-type background (Pearson's Coefficient: ~0.28) (Fig 1D and 1D'). However, in the absence of SID-3, the overlap between hTAC-GFP and 2xFYVE was substantially higher (Pearson's Coefficient: ~0.603), indicating that cargo flow from early endosomes to recycling endosomes was blocked (Fig 1D and 1D'). This observation also indicates that SID-3 modulates recycling at a step similar to that of RAB-10 and EHBP-1 [33].

### Recycling transport remains unaffected in the absence of CDC-42 or ARK-1

Mammalian ACK is recognized as an effector of the Rho family small GTPase CDC42 [16, 17, 24]. Therefore, we investigated the effect of loss of CDC-42 function on endocytic recycling in *C*. *elegans*. Worms homozygous for the putative null allele *cdc-42(gk388)* can only grow to the L3/L4 larval stages [34], this makes acquiring proper intestinal hTAC-GFP images challenging. Hence, we chose to use feeding *cdc-42(RNAi)* bacteria from the Arhinger library [35]. To test the knockdown efficiency of *cdc-42* feeding RNAi, we utilized an animal expressing GFP-CDC-42. Through western blotting and fluorescence imaging, we observed that the *cdc-42* feeding RNAi achieved a significant level of CDC-42 knockdown (S1C and S1C' Fig). To ascertain whether CDC-42/CDC42 directs the function of SID-3 during endocytic recycling, we examined the distribution of hTAC-GFP in CDC-42-knockdown cells (S1C and S1C' Fig). There were no differences in the subcellular localization of hTAC-GFP in CDC-42-deficient cells compared with control cells (Fig 1C–1C''). RME-1 has been extensively studied for its function in endocytic recycling in various tissues [11, 15, 36]. Consistent with the localization of hTAC-GFP, the number of GFP-RME-1-labeled recycling endosomes and the average intensity of GFP-RME-1 were not affected by the lack of CDC-42 (S1D and S1D' Fig) [15]. Moreover, there was only partial overlap between CDC-42 and RAB-10 (Pearson's Coefficient: ~0.51; Mander's Coefficient: ~0.56) (S1E and S1E' Fig). Together these results suggest that SID-3 is likely to act independently of CDC-42 during endocytic recycling in the *C*. *elegans* intestine.

In addition to SID-3, the *C*. *elegans* genome encodes another ACK family protein, ARK-1. To determine whether recycling modulation is a common function of ACK family proteins, we assayed the localization of hTAC-GFP in ARK-1-knockdown animals. However, there was no notable irregularity in the distribution of hTAC-GFP-labeled structures (Fig 1C–1C'').

### SID-3 is located in recycling endosomes

**Fig 2:**
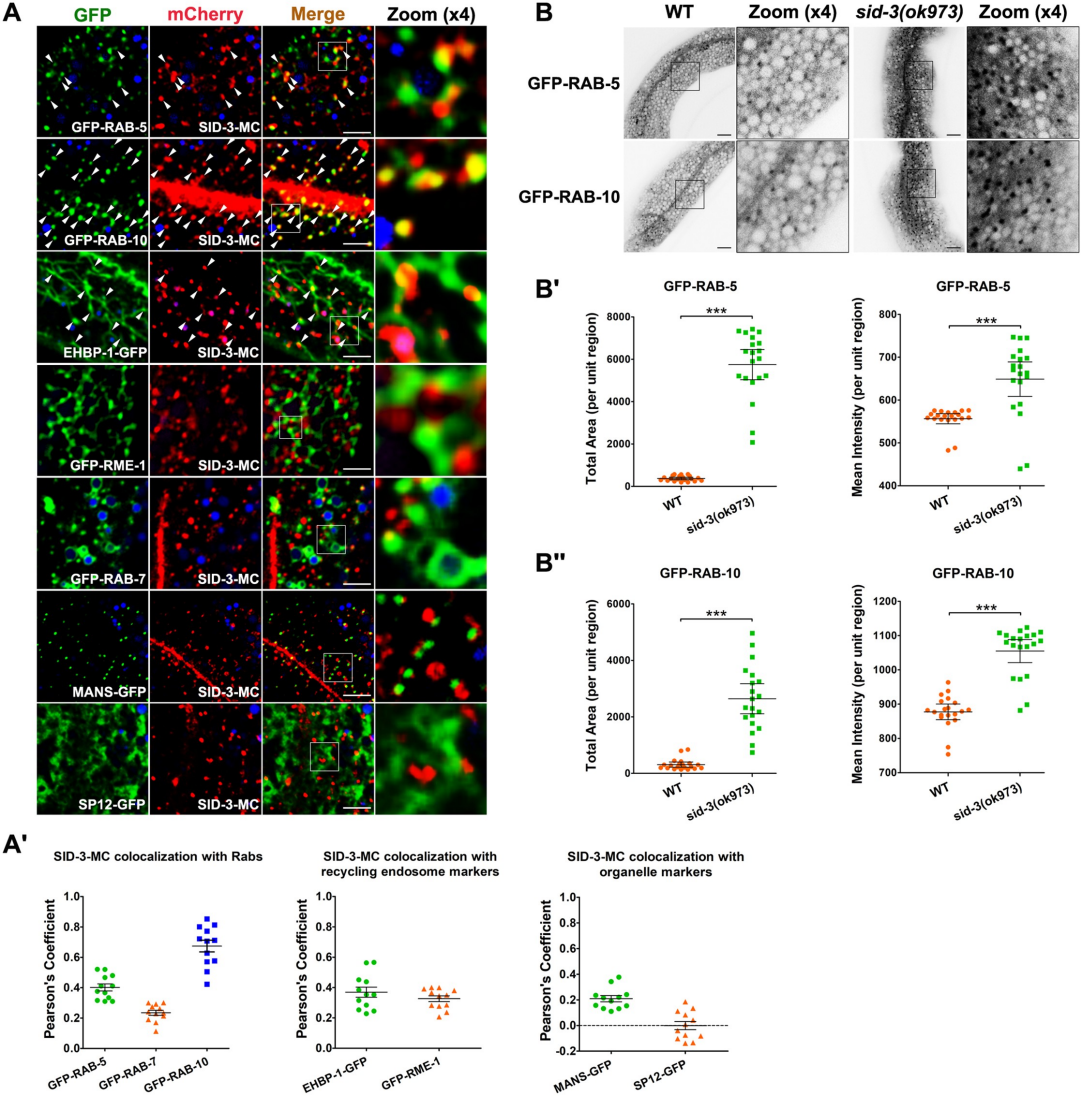
SID-3 localizes in basolateral endosomes. (**A-A'**) Confocal image showing colocalization between SID-3-mCherry and organelle markers in the intestinal cells. SID-3-mCherry colocalizes with early endosomal marker GFP-RAB-5. Also, SID-3-mCherry colocalizes well with GFP-RAB-10. SID-3-mCherry resides in the EHBP-1- and RME-1-labeled recycling endosomal tubules. SID-3-mCherry displayed little colocalization with late endosome marker GFP-RAB-7, Golgi marker MANS-GFP, and ER marker SP12-GFP. Arrowhead indicates a positive overlap. DAPI channel (blue color) indicates broad-spectrum intestinal autofluorescence caused by lipofuscin-positive lysosome-like organelles. Pearson’s correlation coefficients for GFP and mCherry signals are calculated, error bar is 95% CI (n = 12 animals). (**B-Bꞌ**) Confocal images showing early endosomal marker GFP-RAB-5 and sorting endosomal marker GFP-RAB-10 in the intestinal cells. In *sid-3(ok973)* mutants, RAB-5 and RAB-10 labeled structures accumulated on intracellular structures. Error bars are 95% CIs (n = 20 each). Asterisks indicate the significant differences in the Mann-Whitney test (***p<0.001). Scale bars, 10 μm. See S2 Table for quantitative data in this figure.

To define the subcellular localization of SID-3, we compared mCherry-tagged SID-3 with that of a set of well-established organelle markers. In wild-type intestinal cells, RAB-5 resides in punctate early endosomes near the basolateral and apical membranes [33]. We noticed that some puncta labeled by SID-3-mCherry were positive for the early endosome marker GFP-RAB-5 (Pearson's Coefficient: ~0.4) (Fig 2A and 2A'). In the *C*. *elegans* intestine, RAB-10 has been shown to modulate basolateral recycling transport through its function in sorting and recycling endosomes [5, 10]. Notably, prominent colocalization was observed between SID-3 and RAB-10 (Pearson's Coefficient: ~0.67) (Fig 2A and 2A'). EHBP-1 was previously defined as an effector of RAB-10 [12], and is mainly located in tubular and punctate endosomes. Further examination showed that SID-3 often appeared at EHBP-1-positive endosomal tubules (Pearson's Coefficient: ~0.37) (Fig 2A and 2A'). Similarly, SID-3-positive structures were frequently observed on tubules labeled with RME-1 (Pearson's Coefficient: ~0.33) (Fig 2A and 2A'), which is a recycling endosome-associated dynamin-like protein [10, 11, 15, 37]. RAB-7 is known to label small puncta and larger ring-like late endosomes [10]. As expected, SID-3 overlapped with RAB-7 on some punctate structures but was not located on the ring-like late endosomes (Pearson's Coefficient: ~0.23) (Fig 2A and 2A'). We then examined whether SID-3 is localized in the Golgi using a *C*. *elegans* Golgi marker, alpha-mannosidase II-GFP (MANS-GFP) [38], and in the ER using signal peptidase SP12 [38]. However, SID-3 did not colocalize with MANS-GFP (Pearson's Coefficient: ~0.2) or SP12-GFP (Pearson's Coefficient: ~0.09) (Fig 2A and 2A'). In agreement with these observations, SID-3-deficiency had no effects on the distribution and morphology of MANS-GFP-labeled Golgi or SP12-GFP-labeled ER (S2A and S2B Fig). Hence, we concluded that SID-3 mainly resides in sorting and recycling endosomes. Consistent with this notion, loss of SID-3 resulted in a significant increase in the accumulation of RAB-5 (~15-fold increase in total area; ~1.16-fold increase in mean intensity) and RAB-10 (~8.6-fold increase in total area; ~1.2-fold increase in mean intensity) in punctate structures (Fig 2B–2B''). GFP-RAB-11 mainly labels punctate apical recycling endosomes and some puncta that are dispersed in the cytoplasm [10, 39]. Notably, in *sid-3* mutants, there was a moderate increase in the quantity of GFP-RAB-11-labeled structures (~2.4-fold increase in total area), indicating that SID-3 could also be required for apical recycling in intestinal epithelia (S2A and S2B Fig).

### EHBP-1 is required for the endosomal localization of SID-3

**Fig 3:**
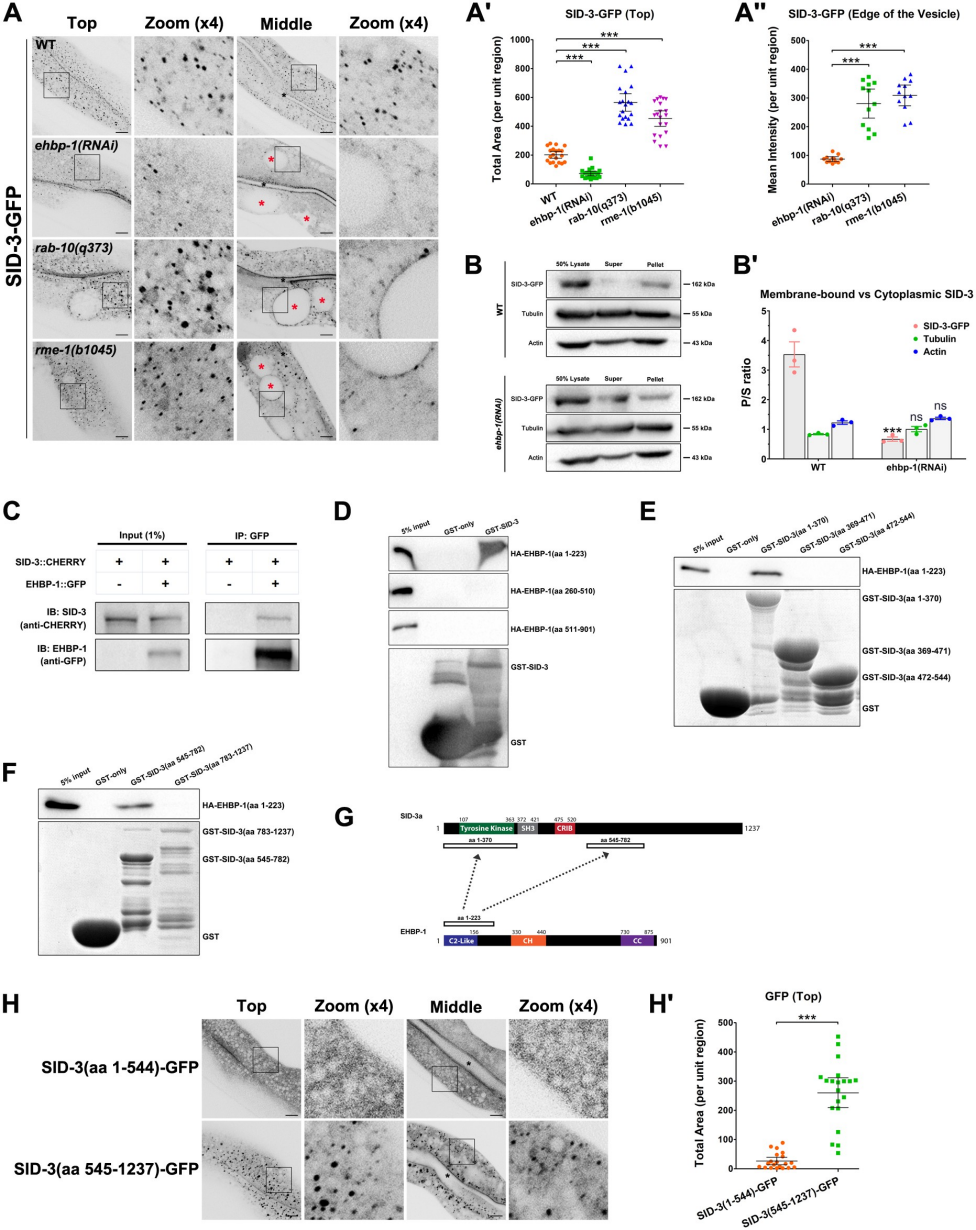
EHBP-1 is required for the endosomal localization of SID-3. (**A-A″**) Confocal images of the worm intestinal cells expressing SID-3-GFP. Black asterisks indicate the intestinal lumen. In the top focal plane, SID-3-GFP labeled punctate structures were diminished in *ehbp-1(RNAi)* animals. In the middle focal plane, SID-3-GFP failed to decorate the edge of enlarged vacuoles. In *rab-10(q373)* or *rme-1(b1045)* animals, SID-3-GFP accumulated on enlarged structures and labeled the vacuoles edges. For A**ꞌ**, error bars are 95% CIs (n = 20 each, 10 animals of each genotype were sampled in whole-cell regions of two intestinal cells). For A**″**, error bars are 95% CIs (n = 12 each, vacuoles edges were manually selected to obtain the fluorescence mean intensity). Asterisks indicate the significant differences in the Mann-Whitney test (***p<0.001). (**B-Bꞌ**) The membrane-to-cytosol ratio of SID-3 decreased in *ehbp-1(RNAi)* animals. Membrane structures were separated from the cytosol of wild-type and *ehbp-1(RNAi)* worm lysates by ultracentrifugation at 100,000xg for 1 hour. SID-3-GFP in the supernatants and pellets were analyzed by western blotting using an anti-GFP antibody. The loading control was blotted by the anti-actin and anti-tubulin antibodies. Quantification of the membrane-to-cytosol ratio (P/S) of SID-3-GFP in wild-type and *ehbp-1(RNAi)* backgrounds. The SEMs from three independent experiments are shown (***p<0.001, ns: no significance). (**C**) Co-immunoprecipitation experiments showing the interaction between SID-3 and EHBP-1. (**D**) Western blot showing GST pulldown with *in vitro* translated HA-tagged proteins. GST-SID-3 interacted with HA-EHBP-1(aa 1–223) while displayed no affinity for other EHBP-1 fragments, including HA-EHBP-1(aa 260–510) and HA-EHBP-1(aa 511–901). (**E**) Western blot showing GST pulldown with *in vitro* translated HA-EHBP-1(aa 1–223). GST-SID-3(aa 1–370) exhibited a prominent interaction with HA-EHBP-1(aa 1–223), and the SH3 domain or CRIB domain failed to display the pulldown band. (**F**) Western blot showing GST pulldown with *in vitro* translated HA-EHBP-1(aa 1–223). GST-SID-3(aa 545–782) interacted with HA-EHBP-1(aa 1–223), and SID-3 C-terminus displayed no affinity for EHBP-1(aa 1–223). **(G)** Schematic diagram of the interactions between SID-3 and EHBP-1, amino acid numbers are indicated. (**H-Hꞌ**) Confocal images showing the distribution of GFP-SID-3(aa 1–544) or GFP-SID-3(aa 545–1237) in the intestinal cells. Truncation containing the C-terminal region (aa 545–1237) was enriched on the punctate structures, while N-terminal fragment containing tyrosine kinase domain, SH3 domain, and CRIB domain (aa 1–544) diffused in the cytosol. Black asterisks indicate intestinal lumen. Error bars are 95% CIs (n = 20 each, 10 animals of each genotype were sampled in whole-cell regions of two intestinal cells). Asterisks indicate the significant differences in the Mann-Whitney test (***p<0.001). Scale bars, 10 μm. See S3 Table for quantitative data in this figure.

To assess the mechanism that determines the endosomal localization of SID-3, we examined the localization of SID-3-GFP in the absence of various recycling regulators. Remarkably, the lack of EHBP-1 led to substantial loss of SID-3 from punctate structures (Fig 3A and 3A'). A previous study showed that *rab-10* and *ehbp-1* mutants accumulated grossly enlarged early/sorting endosomal vacuoles within intestinal cells [10, 12]. Notably, SID-3 accumulated in enlarged endosomes and decorated the vacuoles in RAB-10-deficient cells, but no longer existed on the edges of the endosomal vacuoles labeled with the recycling regulator ARF-6 in *ehbp-1(RNAi)* cells (Fig 3A'', S3A Fig) [40], further supporting the hypothesis that EHBP-1 is required for the endosomal localization of SID-3. Consistent with the labeling of SID-3 on the vacuoles in RAB-10-depleted cells, there was no apparent interaction between SID-3 and the predicted constitutively active (GTPase-defective) form of RAB-10, RAB-10(Q68L) (S3B Fig). To directly evaluate the association of SID-3 with the membrane, we utilized ultracentrifugation to separate the cytosol from the membrane structures in worm lysates and assayed the amount of SID-3-GFP in each fraction by western blotting [41]. As expected, the membrane-to-cytosol ratio of SID-3-GFP in *ehbp-1(RNAi)* animals dropped by ~81% (Fig 3B and 3B'). We also determined whether RME-1 is required for SID-3 endosomal localization. In the *rme-1(b1045)* mutant background, in which intestinal vacuoles mainly consist of enlarged recycling endosomes [10, 15], SID-3-GFP still labeled the recycling endosome vacuoles (Fig 3A–3A''), indicating that the association of SID-3 with endosomes is not dependent on the presence of RME-1.

### Physical association of SID-3 and EHBP-1

Given the requirement of EHBP-1 for the endosomal localization of SID-3, we tested whether EHBP-1 physically interact with SID-3. First, we performed co-immunoprecipitation assays and found that SID-3 associates with EHBP-1 (Fig 3C). To delineate the region of EHBP-1 that interacts with SID-3, we executed a series of *in vitro* binding assays with EHBP-1 truncations [5]. The EHBP-1 lipid-binding C2-like domain (aa 1–223) interacted strongly with SID-3, while the actin-binding CH domain (aa 260–510) and C-terminal coiled-coil domain (aa 511–901) displayed no affinity for SID-3 (Fig 3D and 3G). Further analysis revealed that the C2-like domain of EHBP-1 interacts with the tyrosine kinase domain (aa 1–370) and the central segment (aa 545–782) of SID-3 (Fig 3E–3G).

To investigate the role of different SID-3 domains in endosomal localization, we examined the distribution of GFP-tagged SID-3 truncations (Fig 3H and 3H'). The fragment containing the C-terminal region (aa 545–1237) was enriched in the punctate structures (Fig 3H). Surprisingly, the N-terminal fragment containing the tyrosine kinase, SH3, and CRIB domains (aa 1–544) was diffusely localized in the cytosol (Fig 3H). These results suggest that the central region of SID-3 downstream of the CRIB domain is mainly responsible for endosomal localization.

### The kinase activity of SID-3 is indispensable for endocytic recycling

The SID-3 tyrosine kinase domain is essential for the transmission of RNAi signals [26]. To determine if kinase activity is required for the function of SID-3 in recycling transport, we prepared a kinase-dead form (K139A) and a constitutively active form (L509F) of SID-3 [24, 42]. Like the wild-type SID-3, SID-3(K139A) also localized to EHBP-1-GFP-positive endosomal tubules (Pearson's Coefficient: ~0.35) (S3C and S3C' Fig), indicating that kinase activity is not likely a prerequisite for SID-3 endosomal localization. We then tested the interaction of SID-3(K139A) with the EHBP-1 C2-like domain. SID-3(K139A) displayed a comparable affinity for the C2-like domain as wild-type SID-3 (S3D Fig). Next, we examined the ability of SID-3 mutant forms to complement SID-3-depleted cells. In the middle focal plane, GFP-RME-1 mainly labeled recycling endosomes close to the basolateral plasma membrane and only a limited amount of labeling was observed within the deep cytosol (total area: ~88/unit region) (S3E and S3E' Fig). In *sid-3* mutants, a large number of GFP-RME-1-positive structures accumulated within the cytoplasm (~3.6-fold increase in total area), suggesting that SID-3 is essential for the integrity and distribution of recycling endosomes (S3E and S3E' Fig). Surprisingly, in cells expressing SID-3(K139A), there was no alleviation of intracellular overaccumulation of GFP-RME-1-labeled structures (~4.09-fold increase in total area) (S3E and S3E' Fig). However, the overexpression of SID-3(L509F) fully rescued the cytosolic overaccumulation of GFP-RME-1-positive structures phenotype (S3E and S3E' Fig), further validating the requirement of kinase activity in recycling regulation.

### EHBP-1 sequentially recruits SID-3, NCK-1, and DYN-1

**Fig 4:**
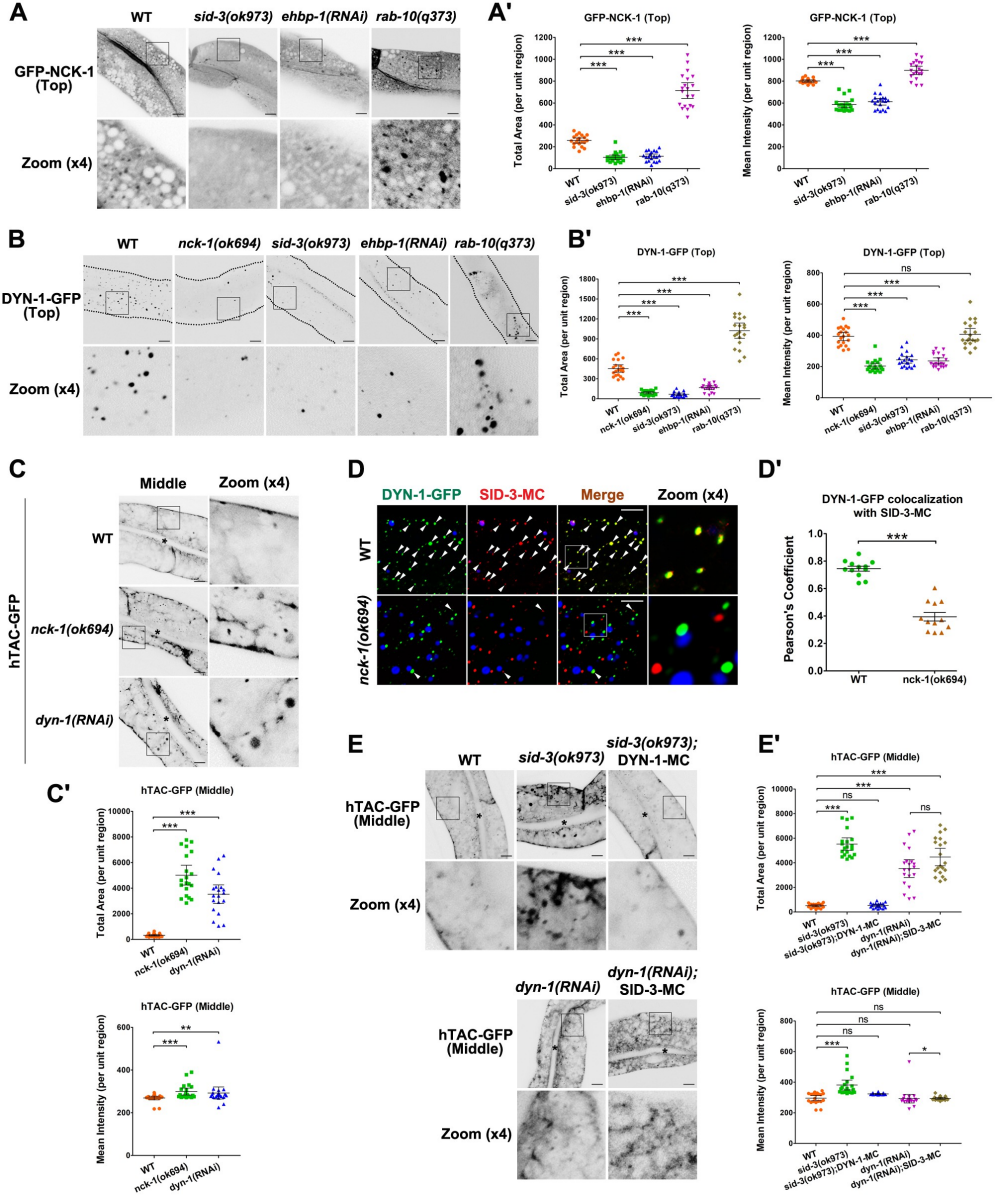
EHBP-1 sequentially recruits SID-3, NCK-1, and DYN-1. (**A-Aꞌ**) Confocal images of the worm intestinal cells expressing GFP-NCK-1. In the top focal plane, GFP-NCK-1 labeled punctate structures. In *sid-3(ok973)* or *ehbp-1(RNAi)* animals, GFP-NCK-1 labeled structures significantly diminished. Instead, GFP-NCK-1 overaccumultaed in punctate structures in *rab-10(q373)* intestinal cells. (**B-Bꞌ**) Confocal images showing DYN-1-GFP subcellular distribution. DYN-1-GFP labeling on intestinal puncta was reduced in *nck-1(ok694)*, *sid-3(ok973)*, and *ehbp-1(RNAi)* intestinal cells. Conversely, DYN-1-GFP overaccumultaed in enlarged structures in *rab-10(q373)* intestinal cells. Boundaries of intestines are outlined by dashed lines. (**C-Cꞌ**) Confocal images showing hTAC-GFP-labeled endosomes in the intestinal cells. In the middle focal plane, hTAC-GFP overaccumulated on the endosomal structures in *nck-1(ok694)* and *dyn-1(RNAi)* cells (P0 adults were scored). Black asterisks indicate intestinal lumen. (**D-Dꞌ**) Confocal image showing colocalization between SID-3-mCherry and DYN-1-GFP in the intestinal cells. SID-3-mCherry colocalized well with DYN-1-GFP in punctate structures. In *nck-1* mutants, the overlap between DYN-1 and SID-3 was reduced. Arrowhead indicates a positive overlap. DAPI channel (blue color) indicates broad-spectrum intestinal autofluorescence caused by lipofuscin-positive lysosome-like organelles. Pearson’s correlation coefficients for GFP and mCherry signals are calculated, error bar is 95% CI (n = 12 animals). (**E-Eꞌ**) Confocal images showing hTAC-GFP-labeled endosomes in the intestinal cells. In the middle focal plane, hTAC-GFP overaccumulated in the endosomal structures in *sid-3(ok973)* and *dyn-1(RNAi)* animals. In the presence of overexpressed DYN-1-mCherry, the accumulation of hTAC-GFP was alleviated. Black asterisks indicate intestinal lumen. Instead, the overexpression of SID-3 could not alleviate the intracellular overaccumulation of hTAC-GFP in *dyn-1(RNAi)* animals. Error bars are 95% CIs (n = 20 each, 10 animals of each genotype were sampled in whole-cell regions of two intestinal cells). Asterisks indicate the significant differences in the Mann-Whitney test (***p<0.001, **p<0.01, *p<0.05, ns: no significance). Scale bars, 10 μm. See S4 Table for quantitative data in this figure.

In *Drosophila*, the SID-3 homolog ACK is required for the intracellular distribution of the adaptor protein Dock/NCK [22]. NCK harbors three SH3 domains and a C-terminal SH2 domain [23], and the interaction with ACK is mediated by the C-terminal SH2 domain [24]. Being a large GTPase, dynamin is capable of assembling into contractile helical polymers that sever the membrane to release the vesicle [43]. Interestingly, dynamin can be co-precipitated with anti-NCK antibody in mammals [25]. Consistent with a previous report [22], we found a significant reduction in GFP-NCK-1-labeled structures in *sid-3(ok973)* mutants (~2.5-fold decrease in total area) (Fig 4A and 4A'), as would be expected if NCK-1/NCK fails to be recruited to endosomes. A similar reduction in DYN-1/dynamin labeling was observed in *sid-3* (~6.5-fold decrease in total area) and *nck-1* (~5.1-fold decrease in total area) mutants (Fig 4B and 4B'). In agreement with the endosomal depletion of SID-3, there was a distinct loss of NCK-1 and DYN-1 from punctate structures in *ehbp-1(RNAi)* animals (NCK-1: ~2.3-fold decrease in total area; DYN-1: ~2.8-fold decrease in total area) (Fig 4A and 4B'). Moreover, in contrast to the vacuoles labeling of NCK-1 in RAB-10-deficient cells (Fig 4A and 4B', S4A–S4A'' Fig), most NCK-1 labeling on the edges of vacuoles was lost in *ehbp-1(RNAi)* and *rab-10(q373);ehbp-1(RNAi)* animals (S4A–S4A'' Fig), indicative of the existence of a protein interaction cascade initiated by EHBP-1. To determine whether NCK-1 and DYN-1 function in the regulation of recycling, we assessed the localization of hTAC-GFP in *nck-1(ok694)* and *dyn-1(RNAi)* animals (Fig 4C and 4C'). As expected, in NCK-1- or DYN-1-depleted cells, the aberrant distribution of hTAC-GFP completely resembled that resulting from a deficit of SID-3 (~11- to ~15-fold increase in total area) (Fig 1C–1C'').

To assay the biochemical interactions between EHBP-1, SID-3, NCK-1, and DYN-1, we performed a series of GST-pulldown experiments. GST-NCK-1 interacted with HA-tagged SID-3(aa 1–544), presumably through its C-terminal SH2 domain (S4B and S4D Fig) [24]. In agreement with a previous study [25], DYN-1 interacted with NCK-1 via its C-terminal region, which includes the pleckstrin homology (PH) domain, GTPase effector domain (GED), and proline-rich domain (PRD) (S4B and S4D Fig). We did not observe an interaction between EHBP-1 and DYN-1 or NCK-1 (S4C Fig), indicating that protein interactions occur sequentially among EHBP-1, SID-3, NCK-1, and DYN-1. Consistent with this, notable colocalization was observed between NCK-1 and SID-3 (Pearson's Coefficient: ~0.66) and DYN-1 (Pearson's Coefficient: ~0.91) in intestinal cells (S4E and S4E' Fig). In the wild-type background, DYN-1 colocalized well with SID-3 (Pearson's Coefficient: ~0.74) (Fig 4D and 4D'). However, in the absence of NCK-1, the overlap between DYN-1 and SID-3 was reduced (Pearson's Coefficient: ~0.39), indicating the interruption of the physical bond between SID-3 and DYN-1 (Fig 4D and 4D').

### Overexpression of DYN-1 alleviates the recycling defects in *sid-3* mutants

Thus far, our findings indicate that the SID-3-NCK-1 complex is a critical link between EHBP-1 and DYN-1. To validate the necessity of DYN-1 for SID-3-mediated recycling transport, we examined the distribution of hTAC-GFP in *sid-3(ok973)* mutants expressing DYN-1-mCherry. The intracellular accumulation of hTAC was substantially reduced by the overexpression of DYN-1 (~10.5-fold decrease in total area) (Fig 4E and 4E'). However, overexpression of SID-3 could not alleviate the intracellular overaccumulation of hTAC-GFP in *dyn-1(RNAi)* animals (~1.26-fold increase in total area) (Fig 4E and 4E'). Because dynamin is known to function in membrane fission through membrane constriction [43, 44], these observations suggest that the SID-3 deficiency-induced disruption of recycling could be due to defective endomembrane fission and the subsequent failure of recycling carrier formation.

### RAB-10 functions upstream of SID-3 in the endocytic recycling pathway

**Fig 5:**
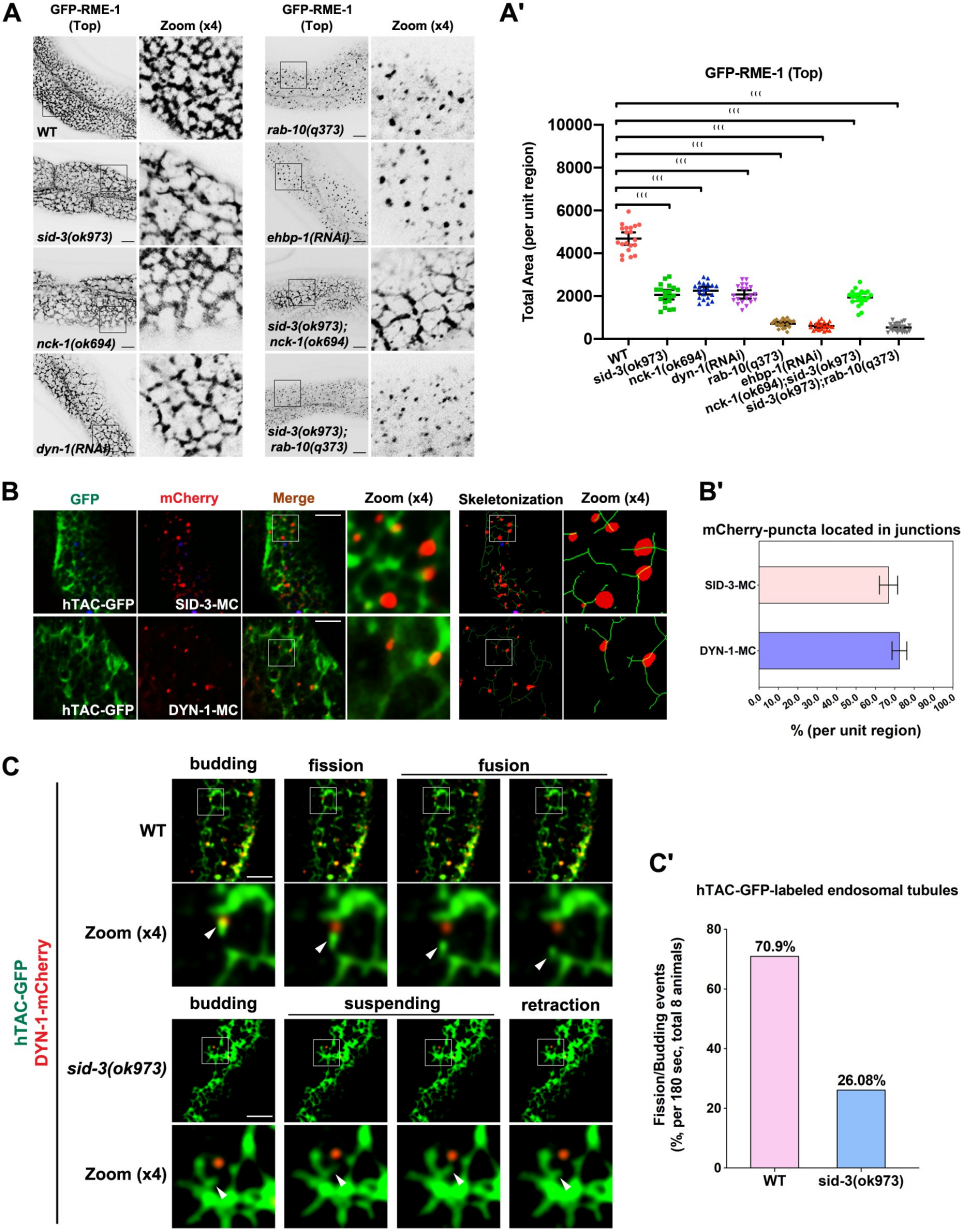
SID-3 is required for endosomal tubules fission. (**A-A'**) Confocal images showing GFP-RME-1-labeled endosomes in the intestinal cells. GFP-RME-1-labeled basolateral (Top) recycling endosomes were reduced in *sid-3(ok973)*, *nck-1(ok694)*, *sid-3(ok973)*;*nck-1(ok694)*, and *dyn-1(RNAi)* (P0 adults were scored) cells. GFP-RME-1-positive endosomal meshwork collapsed in *rab-10(q373)*, *ehbp-1(RNAi)*, and *sid-3(ok973)*;*rab-10(q373)* cells. Error bars are 95% CIs (n = 20 each, 10 animals of each genotype were sampled in whole-cell regions of two intestinal cells). Asterisks indicate the significant differences in the Mann-Whitney test (***p<0.001). (**B-B'**) Confocal images showing the colocalization between hTAC-GFP and SID-3-mCherry or DYN-1-mCherry in the intestines. SID-3 and DYN-1 are located in the hTAC-GFP-labeled tubular endosomes. DAPI channel (blue color) indicates broad-spectrum intestinal autofluorescence caused by lipofuscin-positive lysosome-like organelles. Error bars represent SEMs from the mean (n = 8 each, 8 animals of each genotype were sampled in one unit region of each intestine defined by a 600 x 600 (pixel^2^) box positioned at random). (**C-C″**) Live-cell fluorescence images showing the endosomal network dynamics in *sid-3(ok973)* mutant. The tubules dynamics consisted of following states: budding and/or suspending, fission, and fusion. In a wild-type background, DYN-1-mCherry was positioned at the fission loci of the growing membrane tubules. In *sid-3* mutants, DYN-1-mCherry mislocalized, and the endosomal tubules underwent an extended suspending stage and finally retracted back (8 animals of each genotype were sampled in the intestinal cells). Scale bars, 10 μm. See S5 Table for quantitative data in this figure.

RAB-10 and EHBP-1 work in concert to generate or maintain endosomal structures [5, 12]. Loss of RAB-10 or EHBP-1 led to a notable breakdown of the RME-1-labeled basolateral endosomal meshwork, with tubular organization no longer being maintained (~7-fold decrease in total area) (Fig 5A and 5A'). To elucidate the genetic relationship between RAB-10 and SID-3 in the regulation of recycling endosome morphology, we examined the endosomal architecture in cells lacking both SID-3 and RAB-10. Similar to RAB-10-deficient cells, *sid-3;rab-10* double-mutant cells had punctate structures labeled solely with RME-1, and the endosomal network no longer existed (~8.78-fold decrease in total area) (Fig 5A and 5A'), suggesting that RAB-10 acts upstream of SID-3 in the recycling pathway. In contrast, the pattern of RME-1-labeled structures in *sid-3;nck-1* double mutants, which displayed a moderate reduction in endosomal tubules (~2.4-fold decrease in total area), closely resembled that in *sid-3* mutants (Fig 5A and 5A'). In summary, our results suggest that RAB-10 functions upstream of SID-3 in the recycling pathway, possibly modulating the generation or maintenance of the tubular endosomal network together with EHBP-1.

### Loss of SID-3 diminishes endosomal tubule fission

To better appreciate the mechanism by which SID-3 regulates basolateral recycling, we compared the distribution of hTAC-GFP with that of SID-3-mCherry and DYN-1-mCherry. We found that SID-3 and DYN-1 were often located on hTAC-GFP-positive endosomal tubules (~65% mCherry-puncta near tubular junctions) (Fig 5B and 5B'), prompting us to hypothesize that the SID-3-NCK-1-DYN-1 axis could participate in membrane tubule fission. To test this hypothesis, we tracked endosomal dynamics and noted that membrane tubule dynamics consisted of four stages: budding, suspension, fission, and tubular carrier fusion with the opposite endosomal structures. In the wild-type background, hTAC-GFP-labeled tubules were highly dynamic, with frequent extension and detachment at the DYN-1-enriched endosomal domains (Fig 5C and 5C', S1 Video, ~70.9% fission/budding events). However, in SID-3-depleted cells, the punctate labeling of DYN-1 was greatly reduced. Importantly, the remaining DYN-1-positive puncta rarely overlapped with the tubular recycling endosomes, and the extending tubules often underwent prolonged suspension and finally retracted (Fig 5C and 5C'; S2 Video, ~26.08% fission/budding events).

### Defects in basolateral recycling undermine the competency of feeding RNAi

**Fig 6:**
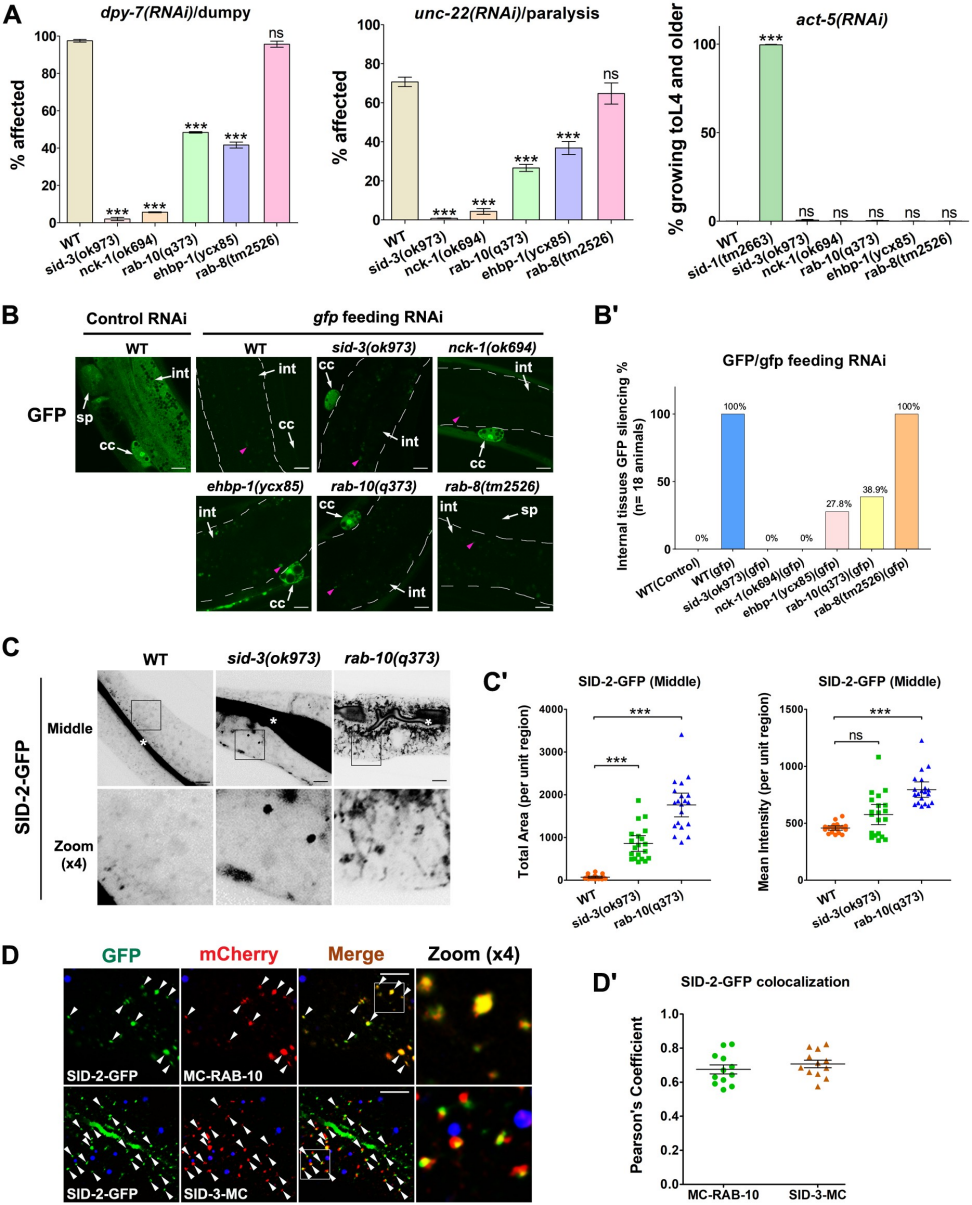
Recycling defect reduces the efficiency of feeding RNAi. (**A**) Feeding RNAi of endogenous genes. Fourth larval stage (L4) animals of wild-type, *sid-3(ok973)*, *nck-1(ok694)*, *rab-10(q373)*, *ehbp-1(ycx85)*, and *rab-8(tm2526)* were fed bacteria expressing dsRNA that target skin (*dpy-7*) and muscle (*unc-22*). The percentage of affected progeny is shown. For feeding RNAi of *act-5* gene, L1 animals were fed bacteria expressing dsRNA that target intestine (*act-5*). Progeny surviving percentage is shown. Error bars are SEMs, n≥100; asterisks indicate the significant differences in the one-tailed Student's t-test (***p<0.001, ns: no significance). (**B-Bꞌ**) Feeding RNAi of GFP in transgenic animals expressing GFP broadly. Synchronized L1 worms in wild-type, *sid-3(ok973)*, *nck-1(ok694)*, *ehbp-1(ycx85)*, *rab-10(q373)*, and *rab-8(tm2526)* genetic backgrounds were fed bacteria expressing *gfp*-dsRNA. White arrows indicate intestine (int), coelomocyte (cc), and spermatheca (sp). Pink arrowheads indicate broad-spectrum intestinal autofluorescence. The percentage of F1 progeny that shows GFP silencing in the coelomocyte and/or spermatheca are quantified (n = 18 animals). (**C-Cꞌ**) Confocal images of the worm intestinal cells expressing GFP-tagged SID-2. White asterisks indicate the intestinal lumen. In *sid-3(ok973)* and *rab-10(q373)* mutants, SID-2-GFP overaccumulated in the intracellular aggregates. Error bars are 95% CIs (n = 20 each, 10 animals of each genotype were sampled in whole-cell regions of two intestinal cells). Asterisks indicate the significant differences in the Mann-Whitney test (***p<0.001). (**D-Dꞌ**) Confocal image showing colocalization between SID-2-GFP and mCherry-tagged RAB-10 or SID-3 in the intestinal cells. SID-2-GFP colocalized well with mCherry-RAB-10. Also, SID-2-GFP and SID-3-mCherry had a significant overlap in intracellular puncta. DAPI channel (blue color) indicates broad-spectrum intestinal autofluorescence caused by lipofuscin-positive lysosome-like organelles. Pearson’s correlation coefficients for GFP and mCherry signals are calculated, error bar is 95% CI (n = 12 animals). Arrowheads indicate a positive overlap. Scale bars, 10 μm. See S6 Table for quantitative data in this figure.

Genetic analysis showed that lack of SID-3 leads to impaired RNAi efficiency [26]. Here we demonstrated that SID-3 plays a significant role in basolateral recycling. Thus, it is of interest to determine whether the reduced feeding RNAi efficiency in *sid-3* mutants is due at least in part to the impairment in recycling. Moreover, if this assumption is valid, the loss of recycling regulators may cause feeding RNAi defects. To rigorously test this possibility, we assayed the effects of feeding RNAi of genes expressed in the skin (*dpy-7*) and muscle (*unc-22*) [26]. Remarkably, *nck-1* mutants exhibited a silencing defect similar to that of *sid-3* mutants (Fig 6A, ~2–6% affected). Likewise, EHBP-1- or RAB-10-depletion resulted in prominent defects in the silencing of *dpy-7* and *unc-22* genes; however, the degree of silencing was much higher compared with that in the *sid-3* and *nck-1* mutants (Fig 6A, ~40–50% affected for *dpy-7*, ~30–40% for *unc-22*). This suggests that, unlike EHBP-1 or RAB-10, SID-3 and NCK-1 are involved in multiple mechanisms underlying the transmission of ingested dsRNA. Indeed, our colocalization study showed that SID-3 was also located in RAB-7-labeled punctate endosomes (Fig 2A), suggesting that SID-3 might be involved in multivesicular body/late endosome-assisted dsRNA export [45–47]. In *C*. *elegans*, RAB-10 and its closest paralog RAB-8 are the homologs of yeast Sec4p. RAB-8 has been implicated in apical exocytosis [10, 48], and RAB-10 and RAB-8 colocalize extensively in endosomes in intestinal cells, suggesting that these endosomes are involved in the sorting of basolateral and apical cargos [10, 12]. However, we did not observe a distinct *dpy-7* or *unc-22* RNAi silencing defect in *rab-8* mutant animals (Fig 6A), suggesting that this phenotype is associated with basolateral recycling, and apical exocytosis is not involved.

To discriminate whether apical endocytic dysfunction contributes to the inefficiency of feeding RNAi in recycling regulator mutants, we examined the effect of silencing of the intestine-expressed gene *act-5* (percentage of surviving progeny), using the *sid-1* mutant as the positive experimental control [26]. SID-1 is a transmembrane protein and functions as a dsRNA-gated channel [49, 50]. Prominently, SID-1 has been postulated to function in releasing endosomal dsRNA into the intestinal cytoplasm after apical endocytosis [51]. Therefore, loss of SID-1 would result in ineffective RNAi silencing of ACT-5 in the intestinal cells (Fig 6A). Consistent with a previous study [26], silencing of ACT-5 was only mildly defective in *sid-3* mutants (Fig 6A). Similarly, lack of NCK-1, EHBP-1, RAB-10, or RAB-8 did not have an adverse effect on the silencing efficiency of ACT-5 (Fig 6A). Taken together, these observations suggest that SID-3, NCK-1, RAB-8, and recycling regulators are not required for the apical uptake of ingested dsRNA.

To further assess the involvement of basolateral recycling in the efficiency of feeding RNAi, we prepared a transgenic strain that broadly expressed GFP and measured the extent of GFP silencing in response to *gfp* feeding RNAi. In a wild-type background, GFP silencing was effective in various tissues, including the intestine, coelomocytes (macrophage-like scavenger cells in the body cavity), and spermatheca (sperm storage organ) (Fig 6B and 6B'). Nevertheless, although silencing still occurred in intestines, loss of SID-3 resulted in a substantial decrease in silencing in coelomocytes. A comparable phenotype was observed in *nck-1*, *ehbp-1*, and *rab-10* mutants (Fig 6B and 6B'), validating the requirement of basolateral recycling for ingested RNAi signal transmission. The closest paralog of RAB-10, RAB-8, is specifically involved in apical exocytosis in intestinal epithelia [48]. We observed extensive GFP silencing in *rab-8* mutants, further demonstrating the necessity and specificity of basolateral recycling during feeding RNAi (Fig 6B and 6B').

### SID-2 is trapped in endosomal structures of recycling mutants

SID-2 interacts with negatively charged dsRNAs and directs the apical endocytosis of ingested dsRNAs from the intestinal lumen [51, 52]. In addition, analysis of SID-1 mosaic animals suggested that ingested dsRNAs could be transported to the pseudocoelom, likely via endosomal transport [51, 53]. These intriguing results led us to speculate whether endocytosed SID-2 could traffic through the recycling compartments *en route* to the basolateral side. It is worth highlighting that, in addition to being enriched in the apical membrane, SID-2-GFP was also located in scattered intracellular puncta (Fig 6C and 6C'). In *sid-3* mutants, there was significant intracellular accumulation of SID-2-GFP in the punctate structures (~12-fold increase in total area) (Fig 6C and 6C'). Moreover, in the absence of RAB-10 or EHBP-1, SID-2-GFP overaccumulated in cytosolic aggregates (~22 to ~24-fold increase in total area) (Fig 6C and 6C', S5A and S5A' Fig), suggesting that the transport of SID-2 requires the basolateral recycling pathway. In agreement with this observation, SID-2 partially overlapped with RAB-10 (Pearson's Coefficient: ~0.675) and SID-3 (Pearson's Coefficient: ~0.707) in the basolateral endosomal structures (Fig 6D and 6D').

To clarify whether the intracellular accumulation of SID-2 is due to defective apical exocytosis, we assayed the transport of the Golgi-derived apical secretory cargo PGP-1-GFP (ATP-binding cassette transporter) [48] in *sid-3(ok973)* cells. The distribution of PGP-1-GFP was normal in *sid-3(ok973)* cells (S5B and S5B' Fig), suggesting that the overaccumulation of SID-2-GFP is not due to apical secretion defects.

## Discussion

**Fig 7:**
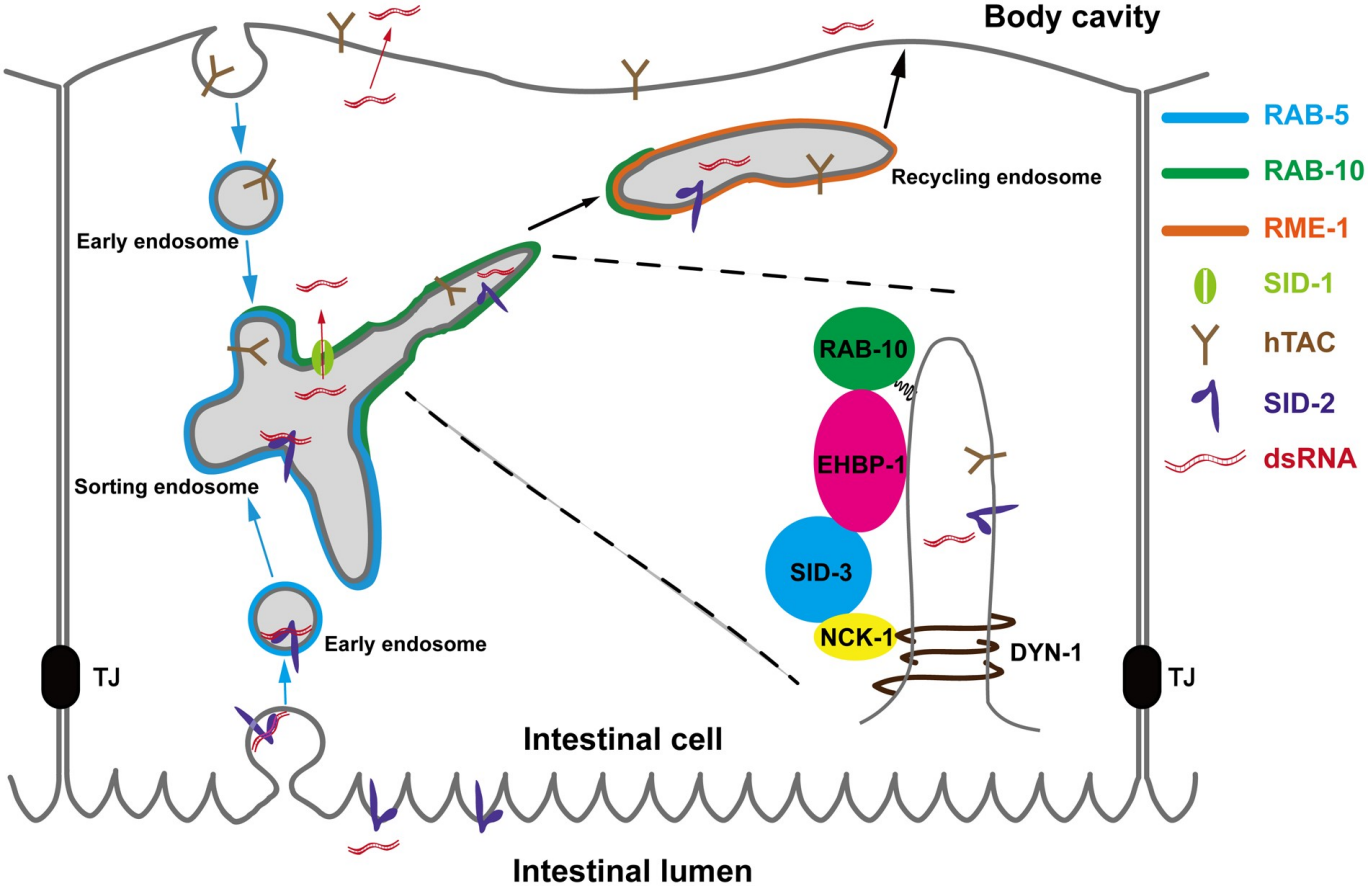
Model of EHBP-1 and SID-3 coordinated recycling in *C*. *elegans* intestine. There is a protein interactive cascade among EHBP-1, SID-3, NCK-1, and DYN-1. SID-3 acts downstream of EHBP-1 to direct recycling endosomal tubules fission in concert with NCK-1 and DYN-1. The dsRNA is internalized from the intestinal lumen via SID-2-mediated endocytosis. Basolateral recycling could be involved in the transport of RNA silencing signals, facilitating the release of apically endocytosed dsRNA into the pseudocoelom (body cavity).

A previous study showed that SID-3 is required for dsRNA endocytosis [26]. Here we identified SID-3 as a novel regulator mediating endocytic recycling in the *C*. *elegans* intestine. SID-3 is localized in basolateral endosomes and participates in an interactive cascade with the RAB-10 effector EHBP-1, NCK-1, and DYN-1. EHBP-1 governs the endosomal localization of SID-3, and SID-3 functions in concert with NCK-1 and DYN-1 to promote the fission of membrane tubules in recycling endosomes (Fig 7).

A series of studies in *C*. *elegans* identified multiple SID proteins involved in systemic RNAi, including SID-1, SID-2, SID-3, and SID-5 [54]. SID-1 and its mammalian homolog SIDT2 have been implicated in the release of endosomal dsRNA into the cytoplasm [51, 55, 56]. Genetic analysis demonstrated that loss of SID-3 leads to impaired RNA mobility, suggesting that SID-3 acts to direct extracellular dsRNA uptake [26]. Accordingly, in mammalian cells ACK/SID-3 is associated with clathrin-coated pits in the plasma membrane [18, 19]. It is worth emphasizing that SID-3 also resides on endosome-like structures in *C*. *elegans* pharynx and intestine [26]. Transgenic overexpression of SID-3 in *sid-3* mutants co-expressing pharyngeal *gfp*-dsRNA resulted in increased silencing of GFP in body-wall muscles and a significant decrease of GFP silencing in the pharynx [26], implying that SID-3 plays a role in dsRNA export. The results of our investigation support this putative function of SID-3. The recycling regulator EHBP-1 recruits SID-3 to basolateral recycling endosomes. SID-3 then cooperates with NCK-1 and DYN-1 to promote membrane tubule fission and subsequent tubular carrier formation, which may facilitate the transport of the ingested RNAi signals to the pseudocoelom. A similar process involving EHBP1L1 and dynamin occurs during apical-directed transport in epithelia [57]. Specifically, the EHBP1L1-Bin1-dynamin complex localizes to the recycling endosomes and promotes the generation of vesicular cargo carriers [57].

In response to *gfp* feeding RNAi, a moderate decrease in nucleus-located SUR-5-GFP silencing was observed in the *sid-3* mutant intestine [26], which was inconsistent with the enhancement of GFP silencing in the pharynx [26]. In this respect, it is noteworthy that *gfp*-dsRNA in the pharynx was derived from pharyngeal transgene expression. In the case of *gfp* feeding RNAi, dsRNA in the intestinal cells could arise from multiple sources, including ingested dsRNA that is apically endocytosed and subsequently released into the cytosol by endosomal SID-1, and dsRNA that is transported to the pseudocoelom and then recaptured via endocytosis [51, 58]. Although there is still a lack of evidence, dsRNA derived from the pseudocoelom has been postulated to contribute to intestinal silencing [58]. Loss of SID-3 leads to trapping of SID-2 in the basolateral endosomes, which may cause a decrease in dsRNA in the pseudocoelom and subsequent defects in silencing in the intestine. An alternative explanation is that disruption of recycling could indirectly impede the preceding apical endocytosis and therefore attenuate RNAi efficiency in intestinal cells. Of note, in contrast to the SUR-5-GFP silencing deficiency observed in *sid-3* mutants, our *gfp* feeding RNAi experiments showed that the efficiency of GFP silencing in *sid-3* mutant intestines was comparable to that in wild-type animals. This phenotypic discrepancy suggests that the *sur-5* gene coding sequence may affect the efficiency of RNA silencing by an as yet to be determined mechanism. Further experiments need to be performed to rigorously test these speculations.

## Materials and methods

### *C*. *elegans* strains and maintenance

All *C*. *elegans* strains were derived from Bristol strain N2 and grown at 20°C on nematode growth media (NGM) plates seeded with *E*. *coli* strain OP50 following standard protocols. A complete list of strains and transgenes (with DNA injection concentrations) used in this study can be found in the S12 Table.

### Feeding RNAi assays

RNAi-mediated silencing was performed following the feeding protocol described by Timmons and Fire [59]. RNAi constructs used in this study were from the Ahringer library [35]. For most experiments, synchronized L1 stage animals were cultured and F1 adults were scored, unless stated otherwise. For the *dpy-7* phenotype, only those animals that were strongly dumpy were counted as dumpy. For the *unc-22* phenotype, only those animals that were incapable of moving upon rapping the plate were scored as paralyzed.

### Genome-wide RNAi screen

Previous studies showed that *rab-10* mutant animals are viable and superficially normal in growth and development [5, 12], and that *rab-10* mutants accumulate the recycling cargo hTAC-GFP in enlarged structures in the deep cytosol of intestinal cells [10]. To identify additional recycling regulators, a large-scale RNAi genetic screen was performed using *rab-10(RNAi)* as the positive control to identify candidate genes whose expression knockdown can lead to the hTAC-GFP overaccumulation phenotype. After the initial screen and follow-up validation, RNAi-mediated knockdown of 54 candidates was found to lead to intracellular hTAC-GFP aggregation, including SID-3, ARX-7, GGTB-1, EXOC-7, HUM-2, and SEC-10. Previous studies indicated that the mammalian homolog of HUM-2/myosin functions with Rab10 to mediate the delivery of GLUT4 storage vesicles to the plasma membrane [60]. SEC-10 and EXOC-7 were implicated in the regulation of hTAC recycling transport in the *C*. *elegans* intestine [8]. *arx-7* encodes a subunit of the Arp2/3 complex, which is also known to be crucial for endocytic trafficking [61]. GGTB-1 is a homolog of human RABGGTB (Rab geranylgeranyltransferase β subunit), which presumably participates in the attachment of a geranylgeranyl group to the cysteine at the C-terminus of Rab [62].

### Antibodies

The following antibodies were used in this study: rabbit anti-actin polyclonal antibody (1:2000, sc-1616-R, Santa Cruz Biotechnologies), rabbit anti-tubulin polyclonal antibody (1:3000, 2148S, Cell Signaling Technology), rabbit anti-HA monoclonal antibody (1:3000, C29F4, Cell Signaling Technology), rabbit anti-GST monoclonal antibody (1:3000, 91G1, Cell Signaling Technology), rabbit anti-GFP polyclonal antibody-Chip Grade (1:2500, ab290, Abcam), and rabbit anti–mCherry polyclonal antibody (1:1000, ab167453; Abcam).

### Protein expression and GST-pulldown assays

The cDNA PCR products of *ehbp-1(1-223aa)*, *ehbp-1(260-510aa)*, *ehbp-1(511-901aa)*, *rab-10(Q68L)*, *sid-3(1-544aa)*, *dyn-1*, *dyn-1(504-838aa)*, and *nck-1* were sub-cloned into a modified *pcDNA3*.*1(+)* vector (Invitrogen, Waltham, MA) with a 2xHA epitope tag and Gateway cassette (Invitrogen, Waltham, MA) for *in vitro* transcription and translation. The cDNA PCR products of *sid-3*, *sid-3(1-370aa)*, *sid-3(369-471aa)*, *sid-3(471-544aa)*, *sid-3(545-782aa)*, *sid-3(545-1042aa)*, *sid-3(783-1237aa)*, *sid-3(1042-1237aa)*, *nck-1*, *nck-1(R326A)*, *ehbp-1*, *nck-1(1-280aa)* and *nck-1(281-395aa)* were transferred in-frame into the vector pGEX-2T (GE Healthcare Life Sciences, Pittsburgh, PA) equipped with a Gateway cassette. The N-terminal HA-tagged proteins were synthesized using the TNT-coupled transcription-translation system (Promega, Madison, WI). Fusion proteins, namely GST-only, GST-SID-3, GST-SID-3(1-370aa), GST-SID-3(369-471aa), GST-SID-3(471-544aa), GST-SID-3(545-1042aa), GST-SID-3(1043-1237aa), GST-SID-3(545-782aa), GST-SID-3(783-1237aa), GST-EHBP-1, GST-NCK-1, GST-NCK-1(R326A), GST-NCK-1(1-280aa) and GST-NCK-1(281-395aa), were expressed in the ArcticExpress strain of *E*. *coli* (Stratagene, San Diego, CA). Bacterial pellets of GST fusions were lysed in 50 mL lysis buffer [50 mM Tris (pH 8.0), 100 mM NaCl, 0.1%NP-40, 10% Glycerol, 1 mM DTT] with Complete Protease Inhibitor Mixture Tablets (Sigma, St. Louis, MO). Extracts were cleared by centrifugation, and supernatants were recovered and incubated with glutathione sepharose 4B beads (GE Healthcare Life Sciences, Pittsburgh, PA) at 4°C for 6 hours. Beads were washed six times with cold washing buffer [50 mM Tris (pH 8.0), 300 mM NaCl, 0.2% (vol/vol) NP-40, 1 mM DTT]. *In vitro*-synthesized HA-tagged protein (15 μL TNT mix diluted in 700 μL Binding buffer) was added to the beads and allowed to bind at 4°C overnight. After six additional washes in washing buffer, the proteins were eluted by boiling in 2× SDS/PAGE sample buffer. Eluted proteins were separated by SDS-polyacrylamide-gel electrophoresis using a 12% (wt/vol) resolving gel and blotted to nitrocellulose or stained with Coomassie blue. After blocking with 5% milk and washing with TBST buffer [20 mM Tris (pH 7.4), 150 mM NaCl, 0.1% (vol/vol) Tween-20], the blot was probed with anti-HA antibody, then stripped and re-probed with anti-GST (91G1) antibody.

### Plasmids and transgenic strains

For expression of GFP or mCherry fusion transgenes specifically in the *C*. *elegans* intestine, two Gateway destination vectors were constructed. The promoter region of the intestine-specific gene *vha-6* or the broadly expressed gene *cdc-42* was cloned into the *C*. *elegans* pPD117.01 vector, followed by the GFP or mCherry coding sequence, a Gateway cassette (Invitrogen, Waltham, MA), the *let-858* 3' UTR sequence, and the *unc-119* gene of *C*. *briggsae*. The cDNA sequences of *pgp-1*, *rme-1*, *mig-14*, *rab-5*, *rab-11*, *SP12* (signal peptidase) [38], *MANS/alpha-mannosidase II* (first 82 aa including the signal sequence/TM-anchor domain) [38], *rab-10*, *rab-7*, *fgt-1*, *dyn-1*, *sid-2*, *sid-3*, *cdc-42*, *nck-1*, *ehbp-1*, *sid-3(K139A)*, *sid-3(L509F)*, *sid-3(545-1237aa)*, *2xfyve* (EEA-1 FYVE motif), *sid-3(1-544aa)*, human *TfR* (transferrin receptor), human *TAC* (alpha-chain of the IL-2 receptor), and *gfp* were introduced into the entry vector pDONR221 by BP reaction and then transferred into the *vha-6* or *cdc-42* promoter-driven vectors in an LR reaction. Complete plasmid sequences are available on request. Low-copy integrated transgenic lines were prepared using the microparticle bombardment method [63]. Additional transgenic lines were obtained using standard microinjection methods (DNA injection concentrations can be found in the Supplementary Table).

### Whole-worm immunoprecipitation

Worms co-expressing SID-3-mCherry and EHBP-1-GFP or expressing only SID-3-mCherry were synchronized and cultured to adulthood on NGM plates. Animals of mixed stages were washed off with M9 buffer (9 cm plates x 10), pelleted and resuspended in ice-cold lysis buffer (25 mM Tris-HCl PH 7.5, 100 mM NaCl, 1 mM EDTA, 0.5% NP-40, 1 mM PMSF, 1 mM Na3VO4, 10 mM NaF and protease inhibitors). The worms were lysed using an automatic grinding machine (Jingxin Inc., Shanghai, China). Carcasses and nuclei were eliminated by centrifugation at 1000xg at 4°C for 10 min, and 500 μl supernatant was incubated with 30 μl GFP-Trap beads (gta-20) (ChromoTek, Munich, Germany) at 4°C for 6 hours. Beads were then washed six times with lysis buffer and proteins were eluted by boiling in 2× SDS/PAGE sample buffer.

### Membrane fractionation assay

Worms expressing intestinal SID-3-GFP were synchronized and cultured on 1 mM IPTG NGM plates seeded with HT115 strains containing a control vector L4440 or *ehbp-1* RNAi vector. Animals of mixed stages were washed off with M9 buffer, and the worm pellet was resuspended in 500 μl lysis buffer (25 mM Tris-HCl PH 7.5, 100 mM NaCl, 1 mM EDTA, 0.5% NP-40, 1 mM PMSF, 1 mM Na3VO4, 10 mM NaF and protease inhibitors). The worms were then disrupted utilizing an automatic grinding machine (Jingxin Inc., Shanghai, China). The lysates were cleared by centrifugation at 1000xg for 20 min at 4°C. The post-cleared lysate (50 μl) was centrifuged at 100,000xg for 1h. Supernatants were collected, and pellets were reconstituted in the same volume of lysis buffer as that of the supernatant. Equivalent amounts of supernatants and pellets were subjected to immunoblotting using anti-actin, anti-tubulin, and anti-GFP antibodies.

### Confocal microscopy

Live *C*. *elegans* animals were mounted on 2% agarose pads with 100 mM levamisole. Multi-wavelength fluorescence images (GFP, mCherry, and DAPI channels) and mono-fluorescence images (GFP channel) were acquired at 20°C using a Nikon C2 laser scanning confocal microscope (Nikon, Tokyo, Japan) equipped with a 100×N.A. 1.2 oil-immersion objective and NIS-Elements AR 4.40.00 software. Z-series of optical sections were obtained using a 0.8-μm step size. For tracking the dynamics of endosomal tubules, animals were loaded into a spinning disc confocal microscope (Olympus IX83, Japan) equipped with a 100x/1.4 oil (WD 013 mm, DIC slider) objective, multiple lasers (405nm, 488nm, 561nm) with the corresponding filters and EMCCD and scMOS cameras. In the *C*. *elegans* intestine, a polarized epithelial tube, the apical membrane faces the lumen, and the basolateral membrane faces the pseudocoelom. The imaging plane close to the basal membrane was defined as “Top”, and the imaging plane where the apical membrane and lumen can be observed was defined as “Middle” (deep cytosol of the intestinal cells) (Fig 1B). Synchronized young adult animals (24 hours after the L4 stage) were used for mono-fluorescence imaging (GFP channel). Synchronized L3 stage animals were selected for multi-wavelength fluorescence imaging (GFP, mCherry, and DAPI channels) and spinning disc dynamics imaging (GFP and mCherry channels). Images taken in the DAPI channel (blue color) were used to identify broad-spectrum intestinal autofluorescence caused by lipofuscin-positive lysosome-like organelles [10].

### Imaging analysis

Fluorescence data from the GFP channel were analyzed by Metamorph software version 7.8.0.0 (Universal Imaging, West Chester, PA). The “Integrated Morphometry Analysis” module of Metamorph was used to measure the fluorescent intensity (mean intensity) and fluorescence area (total area) within unit regions (automatic local background subtraction). From a total of 10 animals of each genotype, “mean intensity” and “total area” were sampled in whole-cell regions of a total of 20 intestinal cells picked at random. In this study, “total area” was used as a composite index of endosomal size and quantity. Typical endosomes cover less area than endosomal structures that overaccumulate in the cytosol. Colocalization images were collected using the open-source Fiji (Image J) software [64]. An object-based plugin, JACoP (Just Another Co-localization Plugin), was used to evaluate Pearson’s correlation coefficients for GFP and mCherry signals; the entire imaging area of 12 animals for each genotype was analyzed.

### Statistical analysis

The significance of differences and 95% confidence intervals were assessed and plotted using Prism software version 5.01 (GraphPad Software, La Jolla, CA). All quantitative data underlying the graphs can be found in the supporting information files.

## Supporting information

S1 Fig (**A-A'**) Confocal images of the worm intestinal cells expressing GFP-tagged recycling cargo protein GFP-FGT-1. In *sid-3(ok973)* mutants, GFP-FGT-1 labeled endosomal structures accumulated within the cytoplasm. Black asterisks in the panels indicate intestinal lumen. (**B-Bꞌ**) Confocal images of the worm intestinal cells expressing GFP-tagged clathrin-dependent recycling cargo hTfR (human transferrin receptor) and clathrin-dependent retrograde cargo MIG-14. In *sid-3(ok973)* mutants, hTfR-GFP and MIG-14-GFP overaccumulated in the plasma membrane. Black asterisks in the panels indicate intestinal lumen. (**C-Cꞌ**) The western blot and fluorescence images showing knockdown efficiency of *cdc-42(RNAi)*. *cdc-42(RNAi)* bacteria feeding achieved a significant level of CDC-42 knockdown in animals expressing the transgenic GFP-CDC-42. (**D-Dꞌ**) Confocal images showing GFP-RME-1-labeled basolateral endosomes in the intestinal cells. Representative images of GFP-RME-1 in wild-type and *cdc-42(RNAi)* animals were obtained. The subcellular distribution of RME-1 was not affected by the loss of CDC-42. Error bars are 95% CIs (n = 20 each, 10 animals of each genotype were sampled in whole-cell regions of two intestinal cells). Asterisks indicate the significant differences in the Mann-Whitney test (*** p<0.001, ns: no significance). (**E-E'**) mCherry-CDC-42 partially overlap with the GFP-RAB-10. DAPI channel (blue color) indicates broad-spectrum intestinal autofluorescence caused by lipofuscin-positive lysosome-like organelles. Pearson’s correlation coefficients for GFP and mCherry signals are calculated, error bars are 95% CIs, n = 12 animals. Scale bars, 10 μm. See S7 Table for quantitative data in this figure.
(TIF)

S2 Fig (**A-B**) Confocal images of the worm intestinal cells expressing GFP-tagged organelle markers. In *sid-3(ok973)* mutants, there was a moderate increase of GFP-RAB-11 labeled apical recycling endosome. Loss of SID-3 had no significant effect on the pattern of MANS-GFP-labeled Golgi or SP12-GFP-labeled ER. Black asterisks in the panels indicate intestinal lumen. Error bars are 95% CIs (n = 20 each, 10 animals of each genotype were sampled in whole-cell regions of two intestinal cells). Asterisks indicate the significant differences in the Mann-Whitney test (*** p<0.001, ns: no significance). Scale bars, 10 μm. See S8 Table for quantitative data in this figure.
(TIF)

S3 Fig (**A**) Confocal images showing that in the absence of RAB-10, SID-3-GFP and ARF-6-mCherry colocalized well at the edges of the vacuoles. In *ehbp-1(RNAi)* animals, SID-3-GFP no longer decorated the vacuoles edges labeled by ARF-6-mCherry. (**B**) Western blot showing GST pulldown with *in vitro* translated HA-RAB-10(Q68L). GST-SID-3 exhibited no interaction with HA-RAB-10(Q68L). (**C-C'**) Confocal image showing colocalization between EHBP-1-GFP and SID-3(K139A)-mCherry in the intestinal cells. SID-3(K139A)-mCherry located at the recycling endosome marker EHBP-1 labeled tubules. DAPI channel (blue color) indicates broad-spectrum intestinal autofluorescence caused by lipofuscin-positive lysosome-like organelles. Arrowheads indicate positive overlap. Pearson’s correlation coefficients for GFP and mCherry signals are calculated, error bar is 95% CI (n = 12 animals). (**D**) Western blot showing GST pulldown with *in vitro* translated HA-EHBP-1(aa 1–223). GST-SID-3(aa 1–370) and GST-SID-3(aa 1–370 K139A) interacted with HA-EHBP-1(aa 1–223). (**E-Eꞌ**) Confocal images showing GFP-RME-1-labeled structures in the intestinal cells. Representative images of wild-type, *sid-3(ok973)*, *sid-3(ok973)*;SID-3-mCherry, *sid-3(ok973)*;SID-3(K139A)-mCherry, and *sid-3(ok973)*;SID-3(L509F)-mCherry animals were obtained. In *sid-3* mutants, hTAC-GFP overaccumulated in enlarged intracellular structures. There was no significant alleviation of hTAC-GFP accumulation upon expression of SID-3(K139A)-mCherry. The overexpression of SID-3(L509F)-mCherry fully rescued the hTAC-GFP accumulation phenotype in *sid-3* mutants. Black asterisks in the panels indicate intestinal lumen. Error bars are 95% CIs (n = 20 each, 10 animals of each genotype were sampled in whole-cell regions of two intestinal cells). Asterisks indicate the significant differences in the Mann-Whitney test (***p<0.001, ns: no significance). Scale bars, 10 μm. See S9 Table for quantitative data in this figure.
(TIF)

S4 Fig (**A-A''**) Confocal images showing GFP-NCK-1 in the intestinal cells. In the middle focal plane, GFP-NCK-1 accumulated on the endosomal vacuoles in *rab-10* mutants. GFP-NCK-1 failed to label the edge of vacuoles in *ehbp-1* mutants. For A**ꞌ**, error bars are 95% CIs (n = 20 each, 10 animals of each genotype were sampled in whole-cell regions of two intestinal cells). For A**″**, error bars are 95% CIs (n = 12 each, vacuoles edges were manually selected to obtain the fluorescence mean intensity). Asterisks indicate the significant differences in the Mann-Whitney test (***p<0.001, ns: no significance). (**B**) Western blot showing GST pulldown with *in vitro* translated HA-tagged SID-3(aa 1–544), DYN-1, and DYN-1(aa 504–838). GST-NCK-1 interacted with HA-SID-3(aa 1–544), HA-DYN-1, and HA-DYN-1(aa 504–838). (**C**) Western blot showing GST pulldown with *in vitro* translated HA-tagged NCK-1 and DYN-1. There was no interaction of GST-EHBP-1 with HA-NCK-1 or HA-DYN-1. (**D**) Schematic diagram of the interactions between SID-3, NCK-1, and DYN-1, amino acid numbers are indicated. (**E-E'**) Confocal image showing colocalization between mCherry-NCK-1 and SID-3-GFP or DYN-1-GFP in the intestinal cells. mCherry-NCK-1 overlapped well with both SID-3-GFP and DYN-1-GFP in punctate structures. Arrowheads indicate positive overlap. DAPI channel (blue color) indicates broad-spectrum intestinal autofluorescence caused by lipofuscin-positive lysosome-like organelles. Pearson’s correlation coefficients for GFP and mCherry signals are calculated, error bar is 95% CI (n = 12 animals). Scale bars, 10 μm. See S10 Table for quantitative data in this figure.
(TIF)

S5 Fig (**A-A'**) Confocal images showing SID-2-GFP in the intestinal cells. In *ehbp-1(RNAi)* animals, SID-2-GFP-labeled structures overaccumulated on enlarged structures. White asterisks in the panels indicate intestinal lumen. (**B-B'**) Confocal images showing PGP-1-GFP in the intestinal cells. In *sid-3(ok973)* mutants, the Golgi-derived apical secretory cargo protein PGP-1-GFP did not exhibit a distribution irregularity. White asterisks in the panels indicate intestinal lumen. Error bars are 95% CIs (n = 20 each, 10 animals of each genotype were sampled in whole-cell regions of two intestinal cells). Asterisks indicate the significant differences in the Mann-Whitney test (*** p<0.001, ns: no significance). Scale bars represent 10 μm. See S11 Table for quantitative data in this figure.
(TIF)

S1 Video hTAC-GFP labeled endosomal dynamics in wild-type animal co-expressing DYN-1-mCherry. Example video of a young adult animal showing 1 frame per 0.7 sec over a 48.3 sec time period.
(AVI)

S2 Video hTAC-GFP labeled endosomal dynamics in *sid-3(ok973)* mutant animal co-expressing DYN-1-mCherry. Example video of a young adult animal showing 1 frame per 0.7 sec over a 12.6 sec time period.
(AVI)

S1 Table Quantitative data of Fig 1. (XLSX)

S2 Table Quantitative data of Fig 2. (XLSX)

S3 Table Quantitative data of Fig 3. (XLSX)

S4 Table Quantitative data of Fig 4. (XLSX)

S5 Table Quantitative data of Fig 5. (XLSX)

S6 Table Quantitative data of Fig 6. (XLSX)

S7 Table Quantitative data of S1 Fig. (XLSX)

S8 Table Quantitative data of S2 Fig. (XLSX)

S9 Table Quantitative data of S3 Fig. (XLSX)

S10 Table Quantitative data of S4 Fig. (XLSX)

S11 Table Quantitative data of S5 Fig. (XLSX)

S12 Table Strain list. A complete list of *C*. *elegans* strains and transgenes (with DNA injection concentration) used in this study.
(XLSX)
