# Supplementary figures and images for: An EHBP-1-SID-3-DYN-1 axis promotes membranous tubule fission during endocytic recycling
Source: PLoS Genet. 2020 May 8;16(5):e1008763. doi: 10.1371/journal.pgen.1008763 (PMC7239482; doi:10.1371/journal.pgen.1008763)

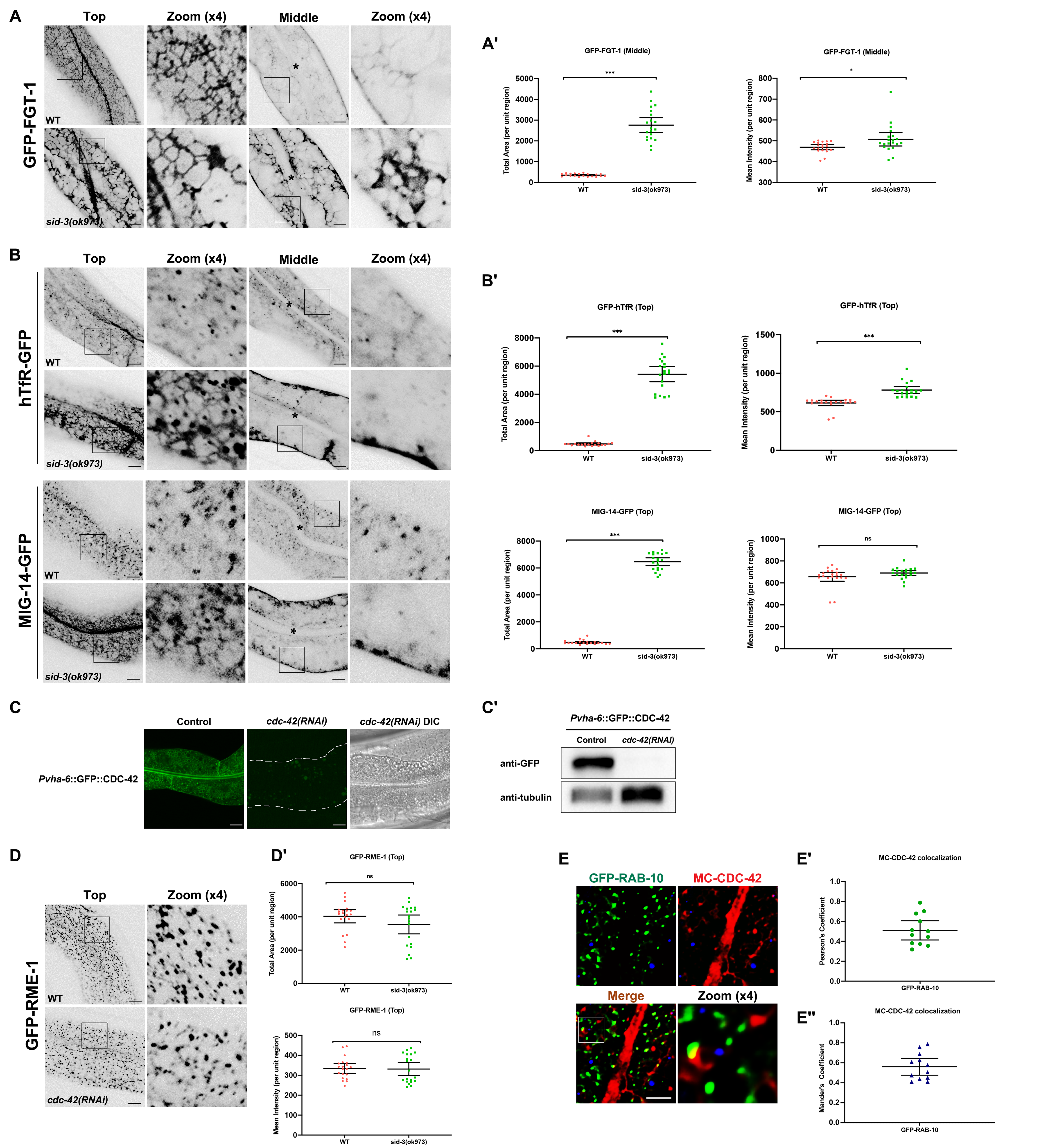

Supplement: S1 Fig — (A-A') Confocal images of the worm intestinal cells expressing GFP-tagged recycling cargo protein GFP-FGT-1. In sid-3(ok973) mutants, GFP-FGT-1 labeled endosomal structures accumulated within the cytoplasm. Black asterisks in the panels indicate intestinal lumen. (B-Bꞌ) Confocal images of the worm intestinal cells expressing GFP-tagged clathrin-dependent recycling cargo hTfR (human transferrin receptor) and clathrin-dependent retrograde cargo MIG-14. In sid-3(ok973) mutants, hTfR-GFP and MIG-14-GFP overaccumulated in the plasma membrane. Black asterisks in the panels indicate intestinal lumen. (C-Cꞌ) The western blot and fluorescence images showing knockdown efficiency of cdc-42(RNAi). cdc-42(RNAi) bacteria feeding achieved a significant level of CDC-42 knockdown in animals expressing the transgenic GFP-CDC-42. (D-Dꞌ) Confocal images showing GFP-RME-1-labeled basolateral endosomes in the intestinal cells. Representative images of GFP-RME-1 in wild-type and cdc-42(RNAi) animals were obtained. The subcellular distribution of RME-1 was not affected by the loss of CDC-42. Error bars are 95% CIs (n = 20 each, 10 animals of each genotype were sampled in whole-cell regions of two intestinal cells). Asterisks indicate the significant differences in the Mann-Whitney test (*** p<0.001, ns: no significance). (E-E') mCherry-CDC-42 partially overlap with the GFP-RAB-10. DAPI channel (blue color) indicates broad-spectrum intestinal autofluorescence caused by lipofuscin-positive lysosome-like organelles. Pearson’s correlation coefficients for GFP and mCherry signals are calculated, error bars are 95% CIs, n = 12 animals. Scale bars, 10 μm. See S7 Table for quantitative data in this figure. (TIF) [file pgen.1008763.s001.tif]

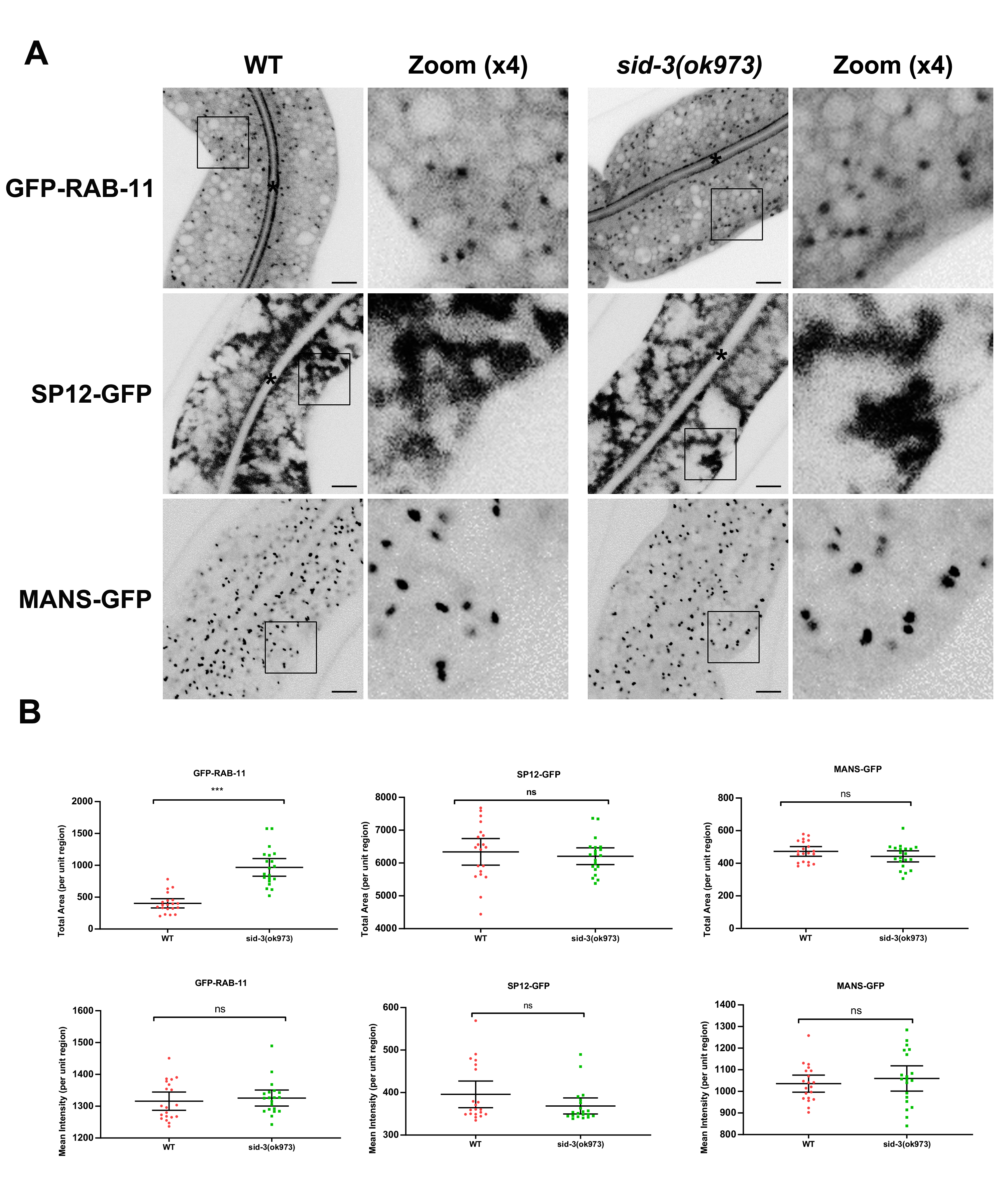

Supplement: S2 Fig — (A-B) Confocal images of the worm intestinal cells expressing GFP-tagged organelle markers. In sid-3(ok973) mutants, there was a moderate increase of GFP-RAB-11 labeled apical recycling endosome. Loss of SID-3 had no significant effect on the pattern of MANS-GFP-labeled Golgi or SP12-GFP-labeled ER. Black asterisks in the panels indicate intestinal lumen. Error bars are 95% CIs (n = 20 each, 10 animals of each genotype were sampled in whole-cell regions of two intestinal cells). Asterisks indicate the significant differences in the Mann-Whitney test (*** p<0.001, ns: no significance). Scale bars, 10 μm. See S8 Table for quantitative data in this figure. (TIF) [file pgen.1008763.s002.tif]

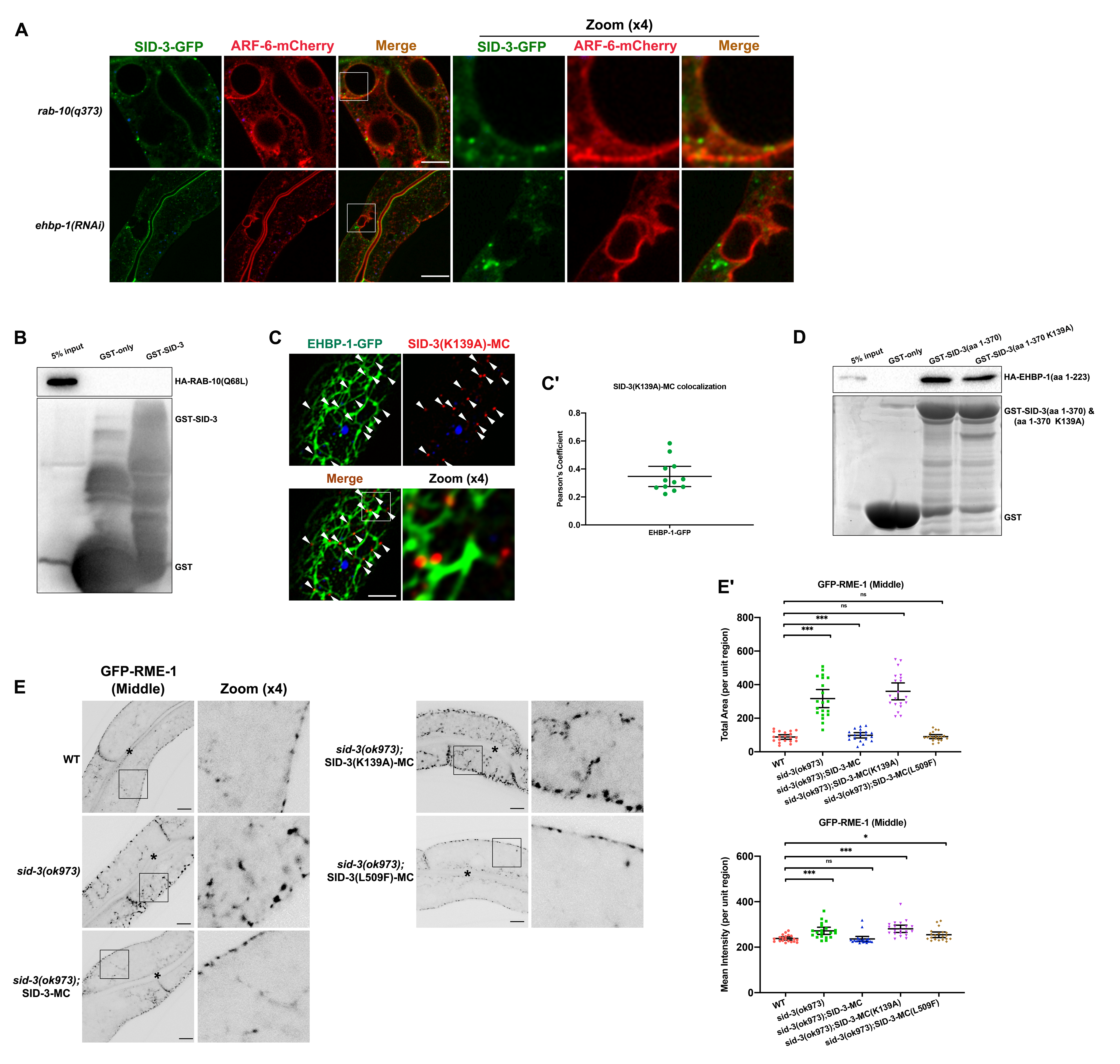

Supplement: S3 Fig — (A) Confocal images showing that in the absence of RAB-10, SID-3-GFP and ARF-6-mCherry colocalized well at the edges of the vacuoles. In ehbp-1(RNAi) animals, SID-3-GFP no longer decorated the vacuoles edges labeled by ARF-6-mCherry. (B) Western blot showing GST pulldown with in vitro translated HA-RAB-10(Q68L). GST-SID-3 exhibited no interaction with HA-RAB-10(Q68L). (C-C') Confocal image showing colocalization between EHBP-1-GFP and SID-3(K139A)-mCherry in the intestinal cells. SID-3(K139A)-mCherry located at the recycling endosome marker EHBP-1 labeled tubules. DAPI channel (blue color) indicates broad-spectrum intestinal autofluorescence caused by lipofuscin-positive lysosome-like organelles. Arrowheads indicate positive overlap. Pearson’s correlation coefficients for GFP and mCherry signals are calculated, error bar is 95% CI (n = 12 animals). (D) Western blot showing GST pulldown with in vitro translated HA-EHBP-1(aa 1–223). GST-SID-3(aa 1–370) and GST-SID-3(aa 1–370 K139A) interacted with HA-EHBP-1(aa 1–223). (E-Eꞌ) Confocal images showing GFP-RME-1-labeled structures in the intestinal cells. Representative images of wild-type, sid-3(ok973), sid-3(ok973);SID-3-mCherry, sid-3(ok973);SID-3(K139A)-mCherry, and sid-3(ok973);SID-3(L509F)-mCherry animals were obtained. In sid-3 mutants, hTAC-GFP overaccumulated in enlarged intracellular structures. There was no significant alleviation of hTAC-GFP accumulation upon expression of SID-3(K139A)-mCherry. The overexpression of SID-3(L509F)-mCherry fully rescued the hTAC-GFP accumulation phenotype in sid-3 mutants. Black asterisks in the panels indicate intestinal lumen. Error bars are 95% CIs (n = 20 each, 10 animals of each genotype were sampled in whole-cell regions of two intestinal cells). Asterisks indicate the significant differences in the Mann-Whitney test (***p<0.001, ns: no significance). Scale bars, 10 μm. See S9 Table for quantitative data in this figure. (TIF) [file pgen.1008763.s003.tif]

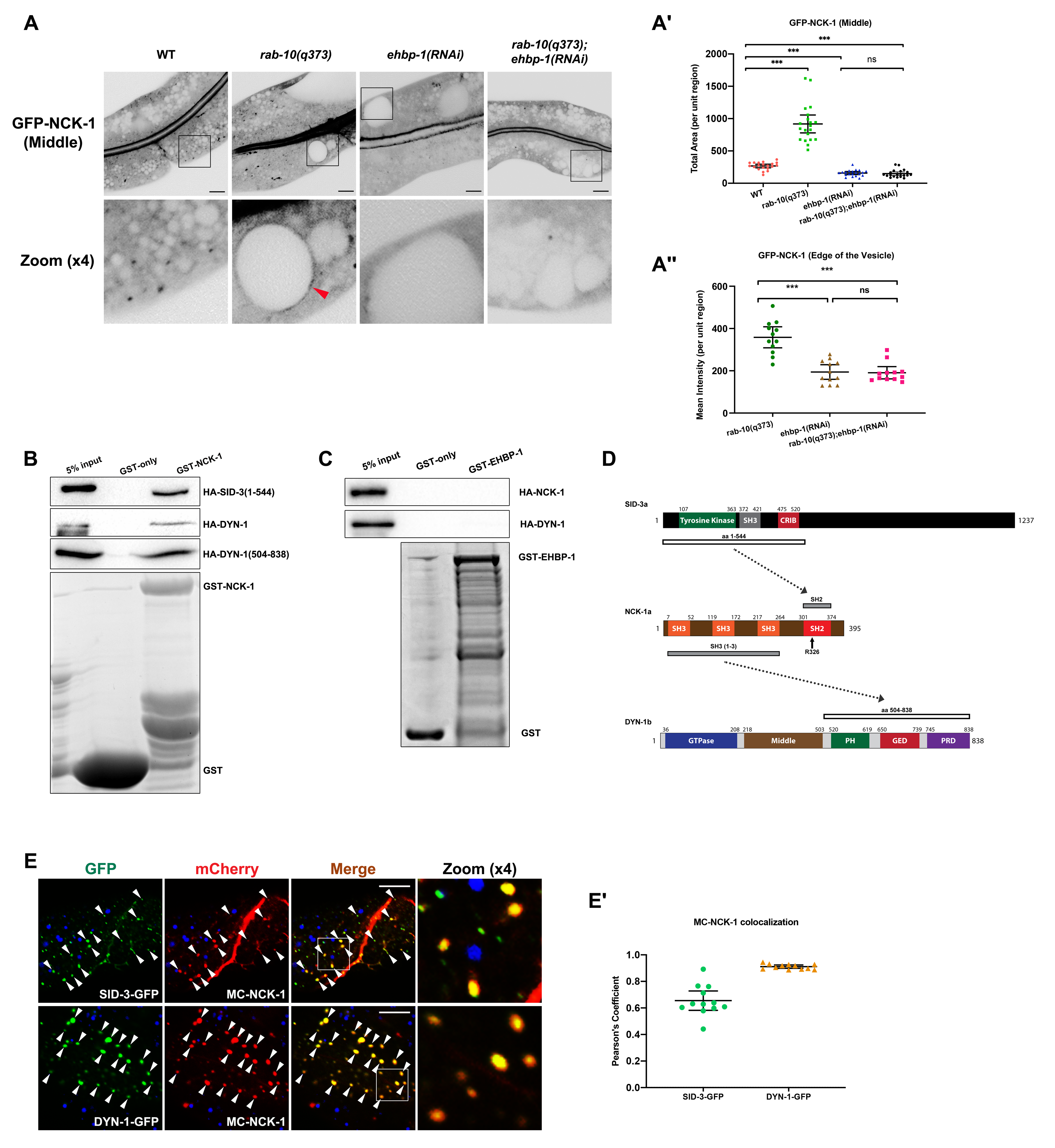

Supplement: S4 Fig — (A-A'') Confocal images showing GFP-NCK-1 in the intestinal cells. In the middle focal plane, GFP-NCK-1 accumulated on the endosomal vacuoles in rab-10 mutants. GFP-NCK-1 failed to label the edge of vacuoles in ehbp-1 mutants. For Aꞌ, error bars are 95% CIs (n = 20 each, 10 animals of each genotype were sampled in whole-cell regions of two intestinal cells). For A″, error bars are 95% CIs (n = 12 each, vacuoles edges were manually selected to obtain the fluorescence mean intensity). Asterisks indicate the significant differences in the Mann-Whitney test (***p<0.001, ns: no significance). (B) Western blot showing GST pulldown with in vitro translated HA-tagged SID-3(aa 1–544), DYN-1, and DYN-1(aa 504–838). GST-NCK-1 interacted with HA-SID-3(aa 1–544), HA-DYN-1, and HA-DYN-1(aa 504–838). (C) Western blot showing GST pulldown with in vitro translated HA-tagged NCK-1 and DYN-1. There was no interaction of GST-EHBP-1 with HA-NCK-1 or HA-DYN-1. (D) Schematic diagram of the interactions between SID-3, NCK-1, and DYN-1, amino acid numbers are indicated. (E-E') Confocal image showing colocalization between mCherry-NCK-1 and SID-3-GFP or DYN-1-GFP in the intestinal cells. mCherry-NCK-1 overlapped well with both SID-3-GFP and DYN-1-GFP in punctate structures. Arrowheads indicate positive overlap. DAPI channel (blue color) indicates broad-spectrum intestinal autofluorescence caused by lipofuscin-positive lysosome-like organelles. Pearson’s correlation coefficients for GFP and mCherry signals are calculated, error bar is 95% CI (n = 12 animals). Scale bars, 10 μm. See S10 Table for quantitative data in this figure. (TIF) [file pgen.1008763.s004.tif]

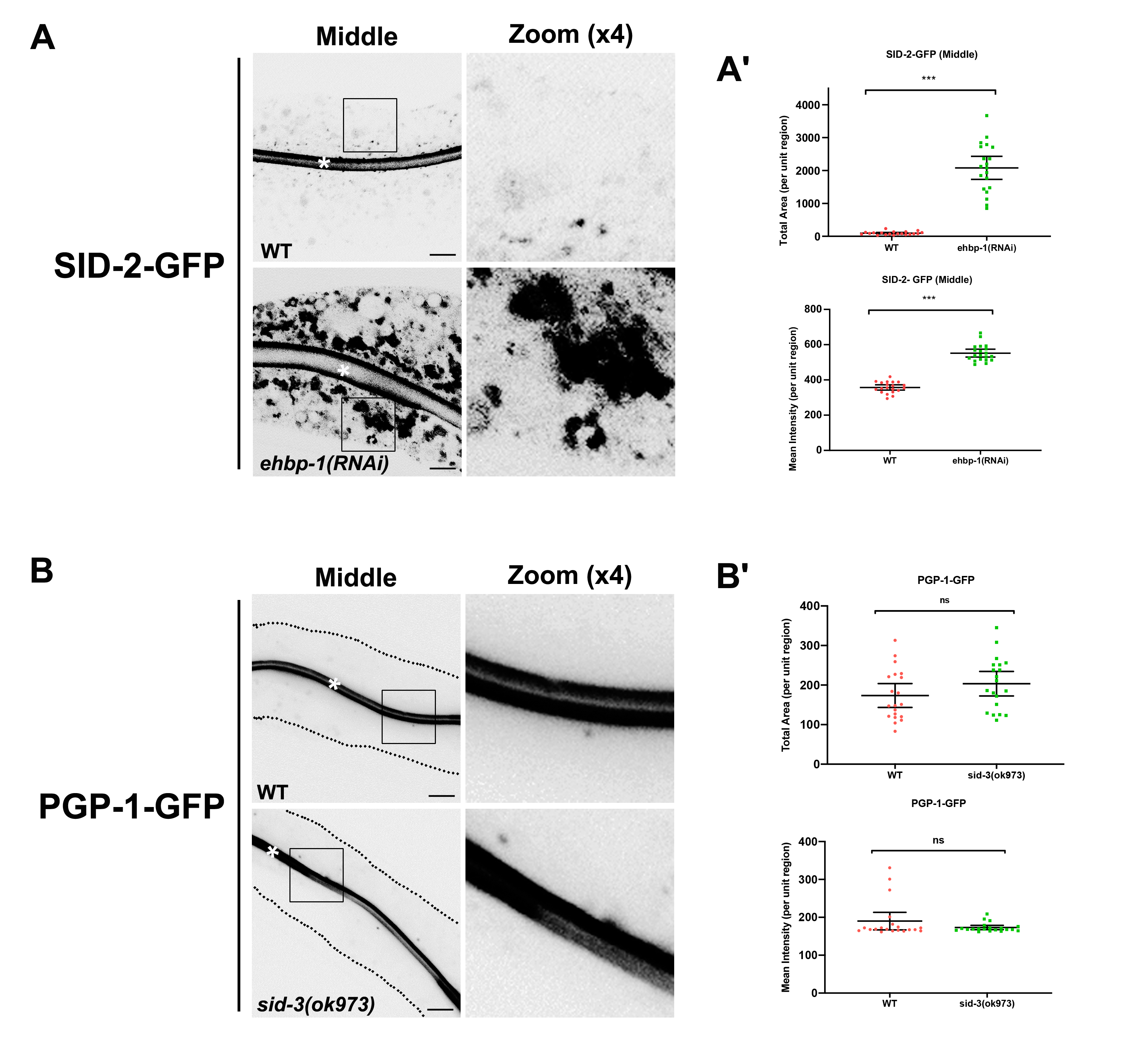

Supplement: S5 Fig — (A-A') Confocal images showing SID-2-GFP in the intestinal cells. In ehbp-1(RNAi) animals, SID-2-GFP-labeled structures overaccumulated on enlarged structures. White asterisks in the panels indicate intestinal lumen. (B-B') Confocal images showing PGP-1-GFP in the intestinal cells. In sid-3(ok973) mutants, the Golgi-derived apical secretory cargo protein PGP-1-GFP did not exhibit a distribution irregularity. White asterisks in the panels indicate intestinal lumen. Error bars are 95% CIs (n = 20 each, 10 animals of each genotype were sampled in whole-cell regions of two intestinal cells). Asterisks indicate the significant differences in the Mann-Whitney test (*** p<0.001, ns: no significance). Scale bars represent 10 μm. See S11 Table for quantitative data in this figure. (TIF) [file pgen.1008763.s005.tif]
